# Single-Cell Transcriptomic Profiling Reveals Regional Differences in the Prefrontal and Entorhinal Cortex of Alzheimer’s Disease Brain

**DOI:** 10.3390/ijms26104841

**Published:** 2025-05-19

**Authors:** Rui-Ze Niu, Wan-Qing Feng, Li Chen, Tian-Hao Bao

**Affiliations:** 1 Mental Health Center, Kunming Medical University, No. 733, Chuanjin Road, Panlong District, Kunming 650034, China; joannacl0417@gmail.com (L.C.); baotianhao@kmmu.edu.cn (T.-H.B.); 2 Laboratory Zoology Department, Kunming Medical University, Kunming 650034, China; fengwanqing123@gmail.com

**Keywords:** Alzheimer’s disease, single-cell transcriptome sequencing, prefrontal cortex, entorhinal cortex, cellular regulatory network

## Abstract

Previous studies have largely overlooked cellular differential alterations across differentially affected brain regions in both disease mechanisms and therapeutic development of Alzheimer’s disease (AD). This study aimed to compare the differential cellular and transcriptional changes in the prefrontal cortex (PFC) and entorhinal cortex (EC) of AD patients through an integrated single-cell transcriptomic analysis. We integrated three single-cell RNA sequencing (scRNA-seq) datasets comprising PFC and EC samples from AD patients and age-matched healthy controls. A total of 124,658 nuclei and 31 cell clusters were obtained and classified into eight major cell types, with EC exhibiting much more pronounced transcriptional alterations than PFC. Through network analysis, we pinpointed hub regulatory genes that form interconnected networks driving AD pathogenesis, findings validated by RT-qPCR showing more pronounced expression changes in EC versus PFC of AD mice. Moreover, dysregulation of the LINC01099-associated regulatory networks in the PFC and EC, showing correlation with AD progression, may present new therapeutic targets for AD. Together, these results suggest that effective AD biomarkers and therapeutic strategies may require simultaneous, precise targeting of specific cell populations across multiple brain regions.

## 1. Introduction

Dementia represents a chronic, acquired, progressive neurocognitive disorder clinically characterized by gradual intellectual decline and varying degrees of personality changes. Current epidemiological statistics reveal about 50 million people with dementia worldwide in 2018, which would triple to 152 million by 2050. Alzheimer’s disease (AD) has emerged as a paramount challenge in aging societies due to the absence of disease-modifying therapies. While a large number of genomic research approaches have identified many AD susceptibility loci associated with clinical symptoms and pathological severity in AD patients [1,2,3], the precise cellular and molecular mechanisms driving AD pathogenesis remain poorly understood.

Recent advances in single-cell technologies, particularly single-nucleus RNA sequencing (snRNA-seq), have revolutionized our capacity to decode the cellular complexity of AD brains. SnRNA-seq enables high-resolution interrogation of cell-type-specific transcriptional networks while overcoming technical challenges associated with intact cell isolation from postmortem brain tissue. Previous hippocampal snRNA-seq studies have already unveiled the presence of disease-associated astrocyte (DAA) and microglial (DAM) subpopulations in AD models, characterized by distinct neuroinflammatory signatures and phagocytic capacities [4,5]. Spatial transcriptomic approaches further revealed amyloid plaque-induced gene networks in peri-plaque microenvironments [6]. Three snRNA-seq studies of human AD cortices have begun mapping region-specific transcriptional alterations [7,8,9], yet critical gaps remain in our understanding of how cellular networks differ across vulnerable brain regions.

The human brain operates as an integrated network of functionally specialized regions, each exhibiting unique cellular compositions and molecular signatures [10]. Different brain regions undertake distinct complex functions determined by cell states, and this regional specialization persists in disease states, potentially explaining the selective vulnerability observed in AD neuropathology. In the disease state, dysregulation of the neural network results in diverse clinical features in patients. A large-scale genomic analysis of 1053 postmortem brain samples from 125 AD patients across 19 cortical regions constructed a region-specific co-expression network associated with AD progression [11] but failed to resolve precisely brain regions specific to AD-related cellular changes.

Here, we presented an integrated snRNA-seq atlas of the prefrontal (PFC) and entorhinal cortex (EC) from AD and control donors and systematically compared eight major cell types across these two regions, including oligodendrocytes, excitatory neurons, oligodendrocyte progenitors, astrocytes, microglia, inhibitory neurons, endothelial cells, and Unknown cells. Pronounced cellular population differences and widespread transcriptomic dysregulation were demonstrated across all cell types, with EC exhibiting 20-fold more differentially expressed genes (DEGs) than PFC regions. Our analysis reconstructed cross-cellular regulatory networks implicating novel molecular pathways in AD pathogenesis, including five LINC01099-associated lncRNA-miRNA-mRNA axes. These findings provide unprecedented resolution into the cellular basis of regional vulnerability in AD, offering new frameworks for understanding disease mechanisms and developing targeted therapeutic strategies.

## 2. Results

### 2.1. Single-Nucleus Transcriptomic Profiling Reveals Region-Specific Cellular Alterations in AD Human Brain Cortex

**Figure 1:**
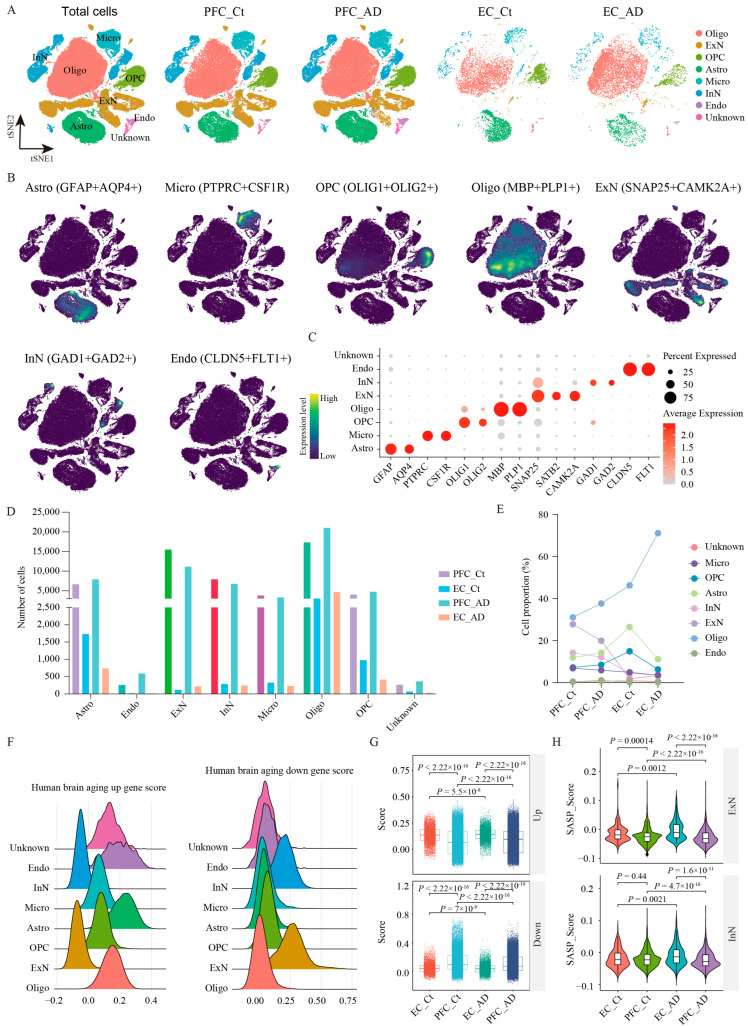
snRNA-seq analysis of PFC and EC in AD patients. (**A**) UMAP plot showing 31 cell clusters obtained after data integration cluster analysis for four groups. (**B**) UMAP plot of the major cell types. (**C**) Dot plot displays the expression of marker genes for each cell type. (**C**) The number of individual cell types in the 124,658 samples. (**D**) The number of individual cell types in PFC_AD, EC_AD, PFC_Ct, and EC_Ct groups. (**E**) Population percentage of eight cell types among different groups. (**F**) Gene set score analysis for eight cell types using human brain aging signatures. (**G**) Gene set score analysis for PFC_AD, EC_AD, PFC_Ct, and EC_Ct groups using human brain aging signatures. (**H**) Gene set score analysis for ExN and InN in different groups using senescence-associated secretory phenotype signature. Two-sided Wilcoxon rank-sum test, FDR < 0.01, log2 (mean gene expression in AD/mean gene expression in control) > 0.25. Color-coded scale bar of gene expression indicates z-scores after normalizing to all the cell types. Oligo: oligodendrocytes; ExN: excitatory neurons; OPC: oligodendrocyte precursor cells; Astro: astrocytes; Micro: microglia; InN: inhibitory neurons; Endo: endothelial cells; Ct: control; PFC: prefrontal cortex; EC: entorhinal cortex.

To obtain a better understanding of the cell-type-specific transcriptomic differences in the PFC and EC of AD patients, we integrated three snRNA-seq datasets containing 30 samples [7,9,12] using Stuart [13]. The integrated dataset included 8 PFC samples from AD patients (PFC_AD), 10 PFC samples from age-matched healthy controls (PFC_Ct), 6 EC samples from AD patients (EC_AD), and 6 EC samples from age-matched healthy controls (EC_Ct) (Table 1 and Table S1). Following stringent quality control, 124,658 nuclei were collected and analyzed (PFC_Ct: 55717, PFC_AD: 55845, EC_Ct: 6533, EC_AD: 6563) after successful batch-effect correction. Unsupervised clustering analysis and t-distributed stochastic neighbor embedding (tSNE) visualization identified eight major cell populations based on the transcriptomic differentially expressed genes (DEGs) and established cell-type unique markers (Figure 1A–C, Figures S1A and S2): *GFAP*^+^*AQP4*^+^ astrocytes (Astro), *CLDN5*^+^*FLT*^+^ endothelial cells (Endo), *SNAP25*^+^*CAMK2A*^+^ excitatory neurons (ExN), *SNAP25*^+^*GAD1*^+^*GAD2*^+^ inhibitory neurons (InN), *PTPRC*^+^*CSF1R*^+^ microglia (Micro), *MBP*^+^*PLP1*^+^ oligodendrocytes (Oligo), and *OLIG1*^+^*OLIG2*^+^ oligodendrocyte precursor cells (OPC), and Unknown cells (lacking specific markers). Comparative analysis revealed distinct region-specific cellular alterations in AD (Figure 1D,E). Astro showed opposing trends, with increased numbers in PFC_AD but decreased numbers in EC_AD versus controls; Micro were reduced in both regions when compared with controls; Oligo increased significantly in both regions; OPC increased in PFC but decreased in EC; neuronal populations (both EnN and InN) were consistently reduced across two regions; and Endo cells and Unknown cells showed no significant quantitative changes (Figure 1D,E).

### 2.2. Brain Aging Exacerbates Regional Vulnerability in AD

Aging is a major risk factor for AD, and its prevalence has become the most pressing challenge to be tackled. To dissect the effects of brain aging on PFC and EC tissues in AD, we performed gene set score analysis using human brain aging signatures and senescence-associated secretory phenotype (SASP) (Figure 1F–H). As revealed, glial cells (Astro, Micro, Oligo, and OPC) showed significantly elevated expression of upregulated aging-associated genes, while neuron populations (ExN and InN) exhibited higher enrichment scores for downregulated aging-associated genes (Figure 1F), suggesting progressive transcriptional silencing with age. Regionally, the EC displayed stronger aging-related dysregulation compared with the PFC in AD. The upregulated aging gene set score was elevated in EC_AD versus PFC_AD, while the downregulated aging score was reduced (Figure 1G). In addition, SASP signatures, indicative of cellular senescence, were markedly higher in EC_AD neurons (both ExN and InN) than in PFC_AD (Figure 1H), reinforcing the EC’s heightened susceptibility to aging. Altogether, our findings demonstrate that brain aging exacerbates regional vulnerability in AD, with neurons showing pronounced transcriptional decline and the EC being more affected than the PFC.

### 2.3. Cellular and Molecular Differences in AD PFC and EC Regions

To comprehensively evaluate the molecular and cellular differences between the PFC and EC in AD, we conducted transcriptomic differential expression analysis and functional enrichment across eight major cell types in both healthy controls and AD patients. Our outcomes revealed distinct region-specific patterns of dysregulation. Interestingly, the functional enrichment of disease-associated DEGs for Astro and OPC exhibited certain similarities between the two regions, while neuronal populations, Micro, and Endo showed substantial differences. Notably, the number of DEGs was consistently higher in all cell types within the EC_AD group than in the PFC_AD group, suggesting more pronounced transcriptomic alterations in the EC during AD pathogenesis and progression. have a more important role in the pathogenesis and development of AD. This observation aligns with the known vulnerability of the EC in early AD pathogenesis and underscores its potential role in driving disease mechanisms.

### 2.4. Region-Specific Dysregulation of Astro in AD

**Figure 2:**
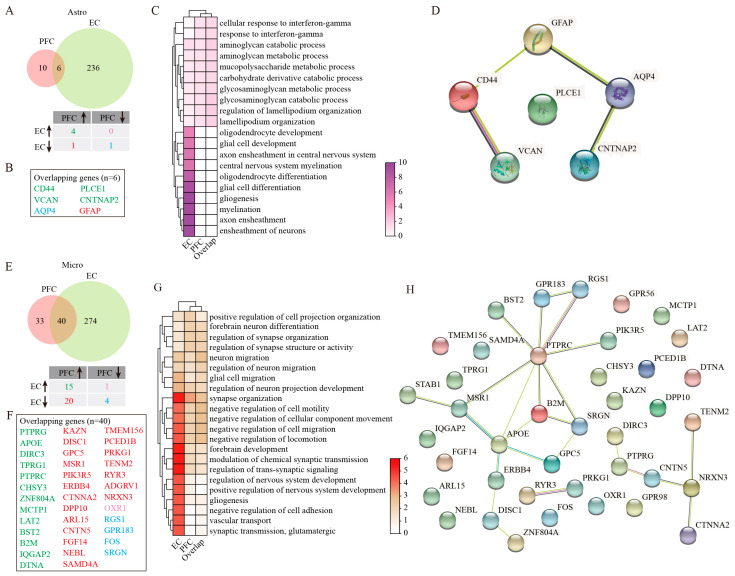
GSEA analysis of DEGs in Astro and Micro. (**A**) Venny plots showing the number of Astro DEGs in PFC and EC of AD patients. (**B**) The overlapping Astro DEGs between PFC and EC regions. (**C**) GO enrichment analysis of Astro DEGs. (**D**) PPI analysis of genes jointly differentially expressed in Astro. (**E**) Venny plots showing the number of Micro DEGs in PFC and EC of AD patients. (**F**) The overlapping Micro DEGs between PFC and EC regions. (**G**) GO enrichment analysis of Micro DEGs. (**H**) PPI analysis of genes jointly differentially expressed in Micro. Two-sided Wilcoxon rank-sum test, FDR < 0.01, log2 (mean gene expression in AD/mean gene expression in control) > 0.25. Color-coded scale bar of gene expression indicates z-scores after normalizing to all the groups.

Astro displayed opposing population trends between the two cortical regions in AD. While their numbers decreased significantly in the EC_AD group compared with healthy controls, they increased in the PFC_AD group (Figure 1D,E). At the molecular level, differential gene expression analysis identified both shared and distinct patterns between two regions, with four genes co-upregulated (*CD44*, *VCAN*, *PLCE1*, and *CNTNAP2*), AQP4 being co-downregulated, and *GFAP* being upregulated in PFC Astro but downregulated in EC Astro (Figure 2A,B, Table S2). GO enrichment analysis revealed that DEGs of EC Astro were predominantly involved in gliogenesis, axon ensheathment, ensheathment of neurons, myelination, glial cell differentiation, oligodendrocyte differentiation, axon ensheathment in the central nervous system, central nervous system myelination, glial cell development, and oligodendrocyte development (Figure 2C), processes critical for neuronal support, and synaptic maintenance. These findings suggest that EC astrocytes may contribute to AD-related cognitive decline through impaired neuron–glia interactions. In contrast, PFC Astro showed no resemblance to EC Astro, with significant enrichment in the aminoglycan catabolic process, glycosaminoglycan catabolic process, regulation of lamellipodium organization, lamellipodium organization, mucopolysaccharide metabolic process, aminoglycan metabolic process, carbohydrate derivative catabolic process, glycosaminoglycan metabolic process, cellular response to interferon-gamma, and response to interferon-gamma (Figure 2C), indicating region-specific inflammatory and metabolic dysregulation. In addition, intersected genes of Astro from two regions further underwent PPI analysis, which demonstrated a core network involving *CD44*, *VCAN*, *AQP4*, *CNTNAP2*, and *GFAP*, with six nodes and four edges (Figure 2D). This network likely represents key molecular players in region-specific Astro responses to AD pathology.

### 2.5. Microglial Heterogeneity Across Two Cortical Regions in AD

Micro populations were reduced in both EC and PFC regions of AD patients relative to healthy individuals (Figure 1D,E), with more pronounced transcriptomic changes in the EC (Figure 2E). Among forty overlapping DEGs between two regions, we identified fifteen consistently upregulated genes (*PTPRG*, *APOE*, *DIRC3*, *TPRG1*, *PTPRC*, *CHSY3*, *ZNF804A*, *MCTP1*, *LAT2*, *BST2*, *B2M*, *IQGAP2*, and *DTNA*) and four downregulated genes (*RGS1*, *GRP183*, *FOS*, and *SRGN*) (Figure 2E,F). Notably, 20 genes were downregulated (*KAZN*, *DISC1*, *GPC5*, *MSR1*, *PIK3R5*, *ERBB4*, *CTNNA2*, *DPP10*, *ARL15*, *CNTN5*, *FGF14*, *NEBL*, *SAMD4A*, *TMEM156*, *PCED1B*, *PRKG1*, *TENM2*, *RYR3*, *ADGRV1*, and *NRXN3*) in the EC but upregulated in the PFC, and OXR1 showed the opposite pattern (Figure 2E,F). Functional enrichment analysis revealed that DEGs of EC Micro were significantly enriched for pathways related to synapse organization, neuron–glia interactions, and neuron migration regulation (Figure 2G). These findings suggested that EC Micro may contribute to AD pathogenesis through defective synaptic pruning and altered neuron–glia communication. However, PFC microglia showed minimal functional enrichment beyond basic synaptic organization, indicating a more limited role in disease progression in this region. PPI analysis of overlapping Micro DEGs from PFC and EC further displayed critical interactions among *PTPRC*, *MSR1*, *APOE*, *B2M*, *SRGN*, and *NRXN3* (Figure 2H). These hub genes likely represent critical regulators of region-specific Micro responses in AD.

### 2.6. Molecular Characterization of Oligo Dysfunction in AD Cortical Regions

**Figure 3:**
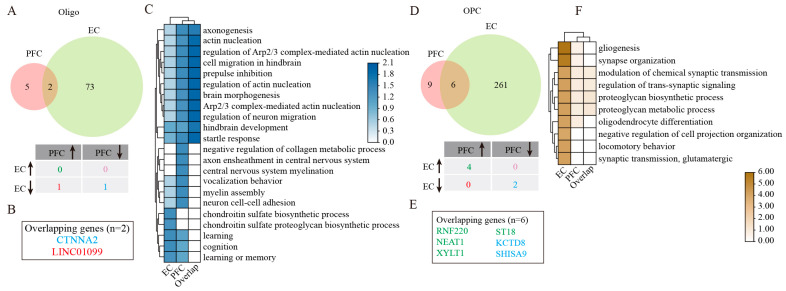
GSEA analysis of DEGs in Oligo and OPC. (**A**) Venny plots showing the number of Oligo DEGs in PFC and EC of AD patients. (**B**) The overlapping Oligo DEGs between PFC and EC regions. (**C**) GO enrichment analysis of Oligo DEGs. (**D**) Venny plots showing the number of DEGs of OPC in PFC and EC of AD patients. (**E**) The overlapping OPC DEGs between PFC and EC regions. (**F**) GO enrichment analysis of DEGs in OPC. Two-sided Wilcoxon rank-sum test, FDR < 0.01, log2 (mean gene expression in AD/mean gene expression in control) > 0.25. Color-coded scale bar of gene expression indicates z-scores after normalizing to all the groups.

Our investigation uncovered a significant increase in Oligo numbers across both the PFC and EC in AD patients compared with healthy controls (Figure 1D,E). Despite this common population trend, molecular profiling uncovered striking regional differences in gene expression patterns. Only two genes overlapped: *LINC01099* (upregulated in PFC, downregulated in EC) and *CTNNA2* (consistently downregulated in PFC and EC) (Figure 3A,B). GO enrichment analysis on DEGs of EC Oligo was mainly associated with learning, cognition, and the chondroitin sulfate proteoglycan biosynthetic process (Figure 3C). These implied Oligo and Astro interactions in the EC could modulate learning, memory, and cognitive function in patients with AD. The overlapping two genes were mainly enriched for regulation of actin nucleation, neuronal migration, and hindbrain development (Figure 3C). Long intergenic non-protein coding RNA 1099 (*LINC01099*) emerged as a particularly interesting candidate. While its biological functions remain to be fully elucidated, genome-wide association studies (GWAS) have linked this molecule to several neurological conditions, including Huntington disease, AD, posterior cortical atrophy, and lipid metabolism regulation (specifically triacylglycerol 46:0 measurement). Through the lncRNASNP2 database prediction [14], we identified 16 potential micro non-coding RNAs (microRNAs) that may interact with LINC01099 (Figure S3A, Table S3). Subsequent functional characterization of these microRNAs using the DIANA-miRPath v3.0 tool [15] revealed their involvement in several critical biological processes, including cellular nitrogen compound metabolic process, biosynthetic process, cellular protein modification process, transcription/DNA-templated, small molecule metabolic process, response to stress, catabolic process, immune system process, and cellular component assembly (Figure S3G). Further KEGG enrichment analysis demonstrated that these miRNAs predominantly participate in multiple signaling pathways regulating the pluripotency of stem cells, Hippo and Wnt signaling pathways, various cancer-related pathways, synaptic plasticity mechanisms (including long-term depression and potentiation), and neurodegenerative processes such as those observed in glioma and TGF-β signaling (Figure S3J).

By integrating data from multiple miRNA target prediction platforms, miRDB [16], miRTarBase [17], and Targetscan [18], we constructed five potential lncRNA-miRNA-mRNA axes that may underlie regional differences in Oligo responses to AD pathology (Supplementary Table S3), including *LINC01099*/*miR-758-3p*/*RORA* (EC_down), *LINC01099*/*miR-146a-5p*/*LRP2* (EC_up), *LINC01099*/*miR-221-23p*/*FOS* (EC_up), *LINC01099*/*miR-222-3p*/*FOS* (EC_up), and *LINC01099*/*miR-377-5p*/*NRXN3* (PFC_up). These findings collectively suggest that *LINC01099* may serve as an important region-specific regulator in AD pathogenesis, potentially exerting its effects through diverse microRNA-mediated mechanisms that differ between cortical areas.

### 2.7. Regional Heterogeneity of OPC Responses in AD

Our analysis of OPC revealed regional dichotomy in AD-related changes, with increased OPC numbers in the PFC and decreased numbers in the EC compared with healthy controls (Figure 1D,E). This opposing pattern was mirrored at the transcriptomic level, with the EC exhibiting a greater number of DEGs than the PFC (Figure 3D). Among these DEGs, we identified six genes that showed consistent regulation patterns across both cortical areas, including four upregulated genes (*RNF220*, *NEAT1*, *XYLT1*, and *ST18*) and two downregulated genes (*KCTD8* and *SHISA9*) (Figure 3D,E). GO functional enrichment analysis provided important insights into the potential roles in region-specific AD pathology. In the EC, OPC DEGs were prominently associated with biological processes related to gliogenesis and Oligo differentiation, synapse organization and neurotransmission, as well as proteoglycan biosynthesis and metabolism (Figure 3F). While OPC in the PFC showed involvement in similar functional categories, the statistical significance of these associations was markedly lower (Figure 3F), suggesting that EC OPC may play a more central role in AD-related pathological processes. This regional disparity in OPC responses may contribute to the differential vulnerability of these cortical areas to AD progression, particularly considering the importance of OPC in maintaining axonal integrity and supporting neuronal function.

### 2.8. ExN Vulnerability and Dysregulation Patterns in AD

**Figure 4:**
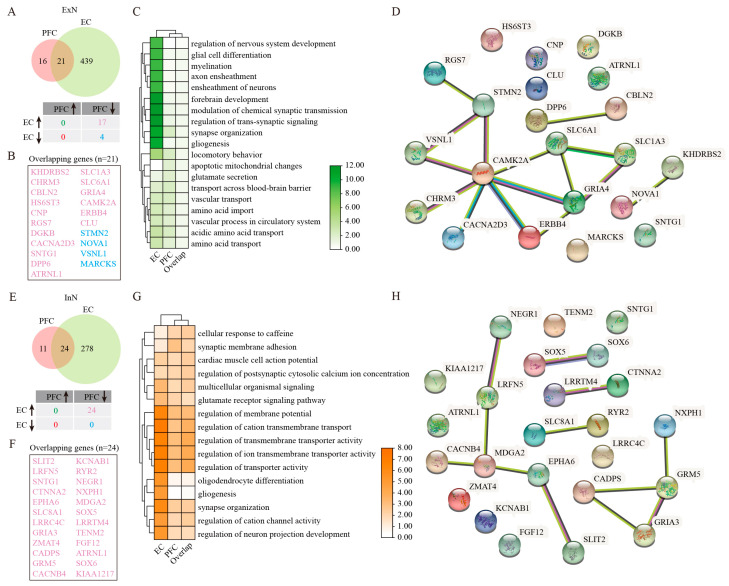
GSEA analysis of DEGs in ExN and InN. (**A**) Venny plots showing the number of DEGs for ExN in PFC and EC of AD patients. (**B**) The overlapping ExN DEGs between PFC and EC regions. (**C**) GO enrichment analysis of DEGs in ExN. (**D**) PPI analysis of genes jointly differentially expressed in ExN. (**E**) Venny plots showing the number of DEGs for InN in PFC and EC of AD patients. (**F**) The overlapping InN DEGs between PFC and EC regions. (**G**) GO enrichment analysis of DEGs in InN. (**H**) PPI analysis of genes jointly differentially expressed in InN. Two-sided Wilcoxon rank-sum test, FDR < 0.01, log2 (mean gene expression in AD/mean gene expression in control) > 0.25. Color-coded scale bar of gene expression indicates z-scores after normalizing to all the groups.

The examination of ExN revealed significant population declines in both cortical regions of AD patients relative to healthy controls (Figure 1D,E). However, transcriptomic analysis demonstrated more extensive gene expression alterations in EC neurons compared with their PFC counterparts (Figure 4A). Among 21 overlapping DEGs identified between two regions, 17 exhibited inverse regulation patterns—downregulated in the PFC but upregulated in the EC (*KHDRBS2*, *CHRM3*, *CBLN2*, *HS6ST3*, *CNP*, *RGS7*, *DGKB*, *CACNA2D3*, *SNTG1*, *DPP6*, *ATRNL1*, *SLC1A3*, *SLC6A1*, *GRIA4*, *CAMK2A*, *ERBB4*, *CLU*), and four genes (*ATMN2*, *NOVA1*, *VSNL1*, *MARCKS*) were downregulated in both two cortical regions (Figure 4A,B). GO enrichment analysis revealed that DEGs of EC ExN showed strong enrichment for biological processes related to synaptic transmission and plasticity, forebrain development, gliogenesis, and myelination (Figure 4C). In contrast, PFC ExN was more associated with pathways involving glutamate transport dynamics, blood–brain barrier function, various vascular processes, amino acid import, and neuron migration (Figure 4C). PPI analysis on overlapping genes displayed several key hub genes, including *CAMK2A* (a critical kinase for synaptic plasticity), *SLC6A1* (a GABA transporter), *GRIA4* (an AMPA receptor subunit), and *STMN2* (a regulator of microtubule dynamics), which formed a network of 21 nodes and 15 edges (Figure 4D), suggesting their potential collective role in mediating region-specific ExN dysfunction in AD.

### 2.9. InN Impairment in AD Cortex

Parallel to ExN, InN populations showed significant reductions in both the PFC and EC of AD patients (Figure 1D,E). Transcriptomic profiling revealed more extensive gene expression changes in EC InN, with all 24 overlapping DEGs (*SLIT2*, *LRFN5*, *SNTG1*, *CTNNA2*, *EPHA6*, *SLC8A1*, *LRRC4C*, *GRIA3*, *ZMAT4*, *CADPS*, *GRM5*, *CACNB4*, *KCNAB1*, *RYR2*, *NEGR1*, *NXPH1*, *MDGA2*, *SOX5*, *LRRTM4*, *TENM2*, *FGF12*, *ATRNL1*, *SOX6*, and *KIAA1217*) displaying inverse regulation patterns (decreased in PFC but increased in EC) (Figure 4E,F). GO functional enrichment analysis demonstrated that DEGs of EC InN were particularly enriched for regulation of cation transmembrane transport, regulation of transporter activity, regulation of ion transmembrane transporter activity, regulation of transmembrane transporter activity, regulation of membrane potential, synapse organization, regulation of neuron projection development, and regulation of cation channel activity (Figure 4G). While PFC InN showed involvement in similar membrane potential regulation pathways, these associations were less extensive and robust compared with the EC (Figure 4G). PPI analysis revealed a statistically significant network (PPI enrichment *p*-value < 1.44 × 10^−8^), comprising 24 nodes and 12 edges, with *GRM5* and *MDGA2* emerging as central interactors (Figure 4H). These findings suggest that region-specific alterations in InN function may contribute differentially to cortical circuit dysfunction in AD, with potentially more pronounced impacts in the vulnerable entorhinal cortex.

### 2.10. Endo Cell Responses to AD Pathology Across Different Brain Regions

**Figure 5:**
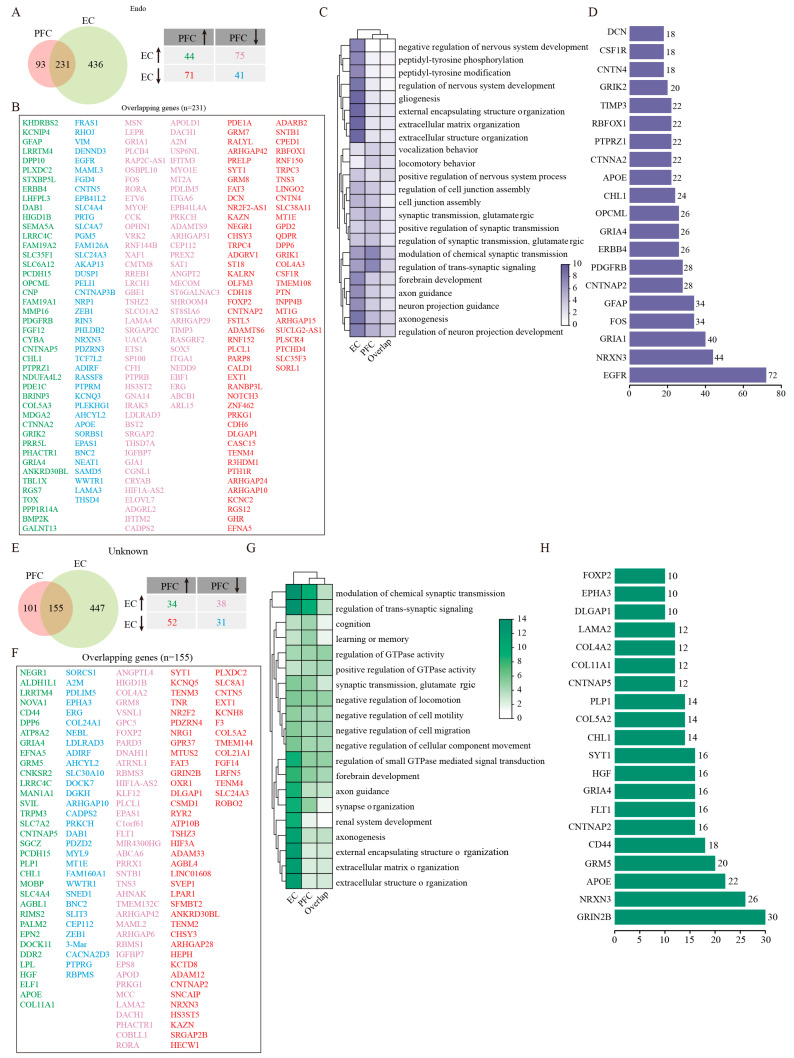
GSEA analysis of DEGs in endothelial and Unknown cells. (**A**) Venny plots showing the number of DEGs of Endo in PFC and EC tissues of AD patients. (**B**) The overlapping Endo DEGs between PFC and EC regions. (**C**) GO enrichment analysis of DEGs in Endo. (**D**) PPI analysis of common DEGs of Endo in PFC and EC of AD patients. (**E**) Venny plots showing the number of DEGs of Unknown cells in PFC and EC of AD patients. (**F**) The overlapping Unknown cell DEGs between PFC and EC regions. (**G**) GO enrichment analysis of DEGs in Unknown cells. (**H**) PPI analysis of common DEGs in Unknown cells. Two-sided Wilcoxon rank-sum test, FDR < 0.01, log2 (mean gene expression in AD/mean gene expression in control) > 0.25. Color-coded scale bar of gene expression indicates z-scores after normalizing to all the groups.

In contrast to other cell types, Endo cell numbers remained relatively stable in both PFC and EC regions of AD patients compared with controls (Figure 1D,E). However, transcriptomic analysis uncovered substantial gene expression changes in the EC, with 231 shared DEGs showing complex regulation patterns between regions (Figure 5A,B). Among these DEGs, 44 genes were upregulated and 41 were downregulated consistently in both regions. While 75 genes downregulated in the PFC were upregulated in the EC, another 71 genes showed the opposite trend (Figure 5A,B). GO enrichment analysis revealed that DEGs of EC Endo were mainly associated with gliogenesis, extracellular matrix organization, and pathways related to regulation of nervous system development, peptidyl-tyrosine phosphorylation, and peptidyl-tyrosine modification (Figure 5C). Whereas PFC Endo demonstrated augmented involvement in synaptic modulation, glutamatergic processes, and cell junction assembly function (Figure 5C). The extensive protein interaction network identified through PPI analysis (comprising 225 nodes and 432 edges) positioned EGFR as a central hub gene (Figure 5D). This finding suggests that epidermal growth factor receptor signaling may play an important role in mediating vascular contributions to AD pathogenesis, potentially through region-specific mechanisms. The preservation of endothelial cell numbers despite significant transcriptomic alterations implies that functional changes in the cerebrovasculature, rather than outright cell loss, may represent an important aspect of AD pathology in these cortical regions.

### 2.11. Characterization of Unknown Cell Populations in AD Cortical Regions

The unidentified cell population, which lacked expression markers characteristic of the seven major defined cell types, showed numerical and DEG patterns similar to endothelial cells across two cortical regions (Figure 1D,E). Likewise, a greater number of DEGs were found in the EC than the PFC, and 155 common DEGs were obtained after the intersection between the two regions (Figure 5E). Of them, 34 genes were upregulated and 31 were downregulated consistently in both PFC and EC. However, 38 genes were upregulated in the EC were downregulated in the PFC, and other 52 genes presented inverse expression in these two regions (Figure 5E,F). Functional enrichment analysis delineated that DEGs of EC Unknown cells were mainly involved in such biological processes as synaptic regulation, extracellular matrix organization, axonogenesis, regulation of small GTPase-mediated signal transduction, and axon guidance (Figure 5G). The biological processes strongly enriched by DEGs of PFC Unknown cells included modulation of chemical synaptic transmission, regulation of trans-synaptic signaling, synapse organization, forebrain development, and regulation of GTPase activity (Figure 5G). Protein interaction network analysis identified GRIN2B as a pivotal node in a network comprising 152 nodes and 181 edges (Figure 5H). This finding raises the intriguing possibility that these Unknown cells may participate in glutamatergic signaling pathways, potentially contributing to region-specific aspects of AD pathophysiology. Further investigation will be required to precisely define the nature and functional significance of this cell population in AD progression.

### 2.12. Comparison Integration of Single-Nucleus and Bulk RNA Sequencing Revealed Conserved Molecular Networks in AD

**Figure 6:**
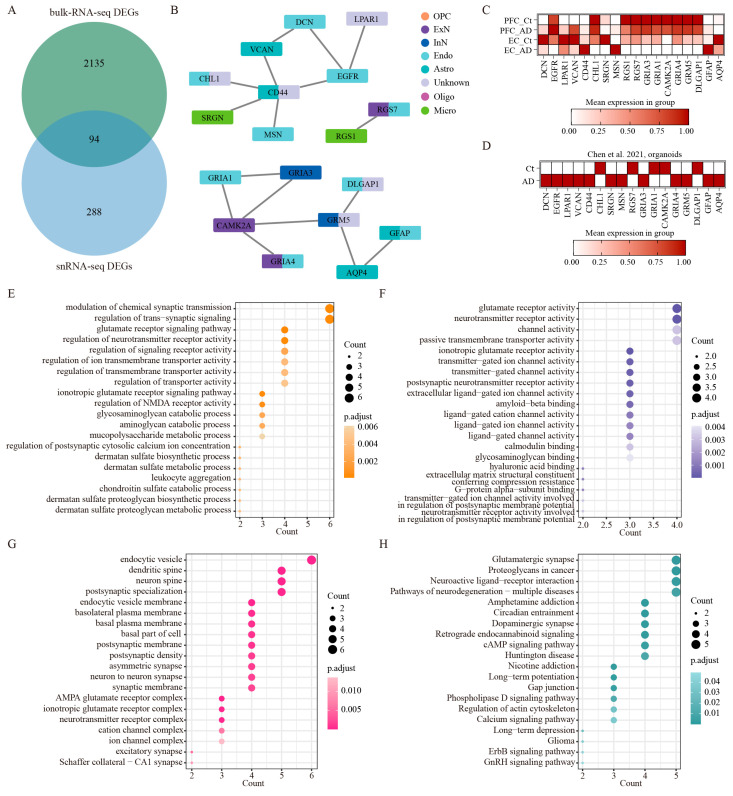
The integrated analysis of bulk RNA-seq and snRNA-seq. (**A**) The overlapped DEGs of bulk RNA-seq (limma moderated t-statistics, false discovery rate (FDR) < 0.01, FC > 1.5) and snRNA-seq (two-sided Wilcoxon rank-sum test, FDR < 0.01, log2 (mean gene expression in AD/mean gene expression in control) > 0.25). (**B**) PPI network of key regulatory genes out of the 94 overlapped genes in the eight cell types. The minimum required interaction score is highest confidence (0.900). (**C**) The expression patterns of key regulatory genes across different brain regions in our integrated human AD snRNA-seq data. (**D**) The expression patterns of the same gene set in published AD organoid single-cell sequencing data [19]. (**E**–**G**) GO enrichment analysis of the overlapped genes between bulk RNA-seq data and snRNA-seq data, BP (**E**), MF (**F**), and CC (**G**). (**H**) Analysis of KEGG signaling on the overlapped genes between bulk RNA-seq data and snRNA-seq data.

To bridge the gap between single-cell resolution and whole-tissue analyses, we performed an extensive integrative analysis comparing snRNA-seq datasets with a large-scale bulk RNA-seq study by Wang M et al. [11] that examined 1053 postmortem brain samples across 19 cortical regions from 125 AD patients. This multi-level comparison yielded 94 significantly overlapping DEGs (FDR-corrected *p* < 0.01 by two-sided Wilcoxon rank-sum test) out of 2229 DEGs from the bulk RNA-seq data and 485 DEGs from our snRNA-seq data (Figure 6A, Table S4). This importantly identifies a core set of conserved molecular changes that are detectable across different technological platforms and analytical approaches. To elucidate the association among overlapped genes in the eight cell types, we constructed a PPI network analysis, which revealed robust interaction relationships among 18 key regulatory genes spanning all eight cell types (highest confidence: 0.9) (Figure 6B, Tables S5 and S6). The expression patterns of these 18 genes in AD patients across the PFC and EC regions were demonstrated using our integrated snRNA-seq data (Figure 6C). Notably, genes including *DCN*, *EGFR*, *LPAR1*, *VCAN*, *CD44*, *CHL1*, *GFAP*, and *AQP4* show differential expression patterns across the two brain regions. *SRGN*, *MSN*, *RGS1*, *RGS7*, *GRIA3*, *GRIA1*, *CAMK2A*, *GRIA4*, *GRM5*, and *DLGAP1* were consistently upregulated or downregulated in these two regions. We further present the expression patterns of the same gene set in an integrated analysis of published AD organoid single-cell sequencing data [19], which demonstrated conserved expression changes for several genes (*CD44*, *CHL1*, *MSN*, *RGS7*, *GRIA1*, *CAMK2A*, *DLGAP1*, *GFAP*) between human brain tissue and organoid models, while also identifying model-specific differences (Figure 6D). Additional functional enrichment analysis demonstrated that the network components participated in several fundamental biological processes that are particularly relevant to AD pathogenesis: synaptic signaling and neurotransmission, glutamatergic signaling, and extracellular matrix processes (Figure 6C, Table S7). The molecular functions enriched by these genes primarily included glutamate receptor activity, neurotransmitter receptor activity, transmitter-gated channel activity, postsynaptic neurotransmitter receptor activity, extracellular ligand-gated ion channel activity, amyloid-beta binding, ligand-gated cation channel activity, and hyaluronic acid binding (Figure 6D, Table S8). At the subcellular level, these genes localized prominently to postsynaptic specializations, including dendritic spines and neuron spines (Figure 6E, Table S9). Interestingly, we also observed enrichment in endocytic vesicles and basolateral plasma membranes (Figure 6E, Table S9), implicating these genes in both synaptic function and cellular trafficking processes. Further KEGG pathway analysis revealed a significant enrichment of these genes in such important signaling pathways as synaptic plasticity pathways, neurodegenerative signaling, and neuromodulatory systems (Figure 6F, Table S10). To validate these findings, we also performed enrichment analysis using the key regulatory genes identified through the aforementioned snRNA-seq datasets. Remarkably, this separate analysis recapitulated nearly identical biological themes to those of 94 overlapped genes. As for biological processes, modulation of synaptic transmission, glutamate receptor signaling pathway, and regulation of neurotransmitter receptor activity were significantly enriched (Figure S4A). The molecular functions enriched by these regulatory genes also primarily included glutamate receptor activity, neurotransmitter receptor activity, and amyloid–beta binding (Figure S4B). The cell components’ outcomes of them unveiled nearly identical cellular localizations (Figure S4C). Meanwhile, such overlapping signaling pathways as glutamatergic synapse, amphetamine addiction, proteoglycans in cancer, circadian entrainment, dopaminergic synapse, long-term potentiation, and neuroactive ligand-receptor interaction were significantly involved by these key regulatory genes (Figure S4D). This strong concordance between different analytical approaches significantly strengthens the biological validity of these findings and suggests we have identified core molecular networks that are consistently altered in AD across multiple dimensions of analysis.

### 2.13. Expression Variation of Key Network Genes in the PFC and EC of AD Mice

**Figure 7:**
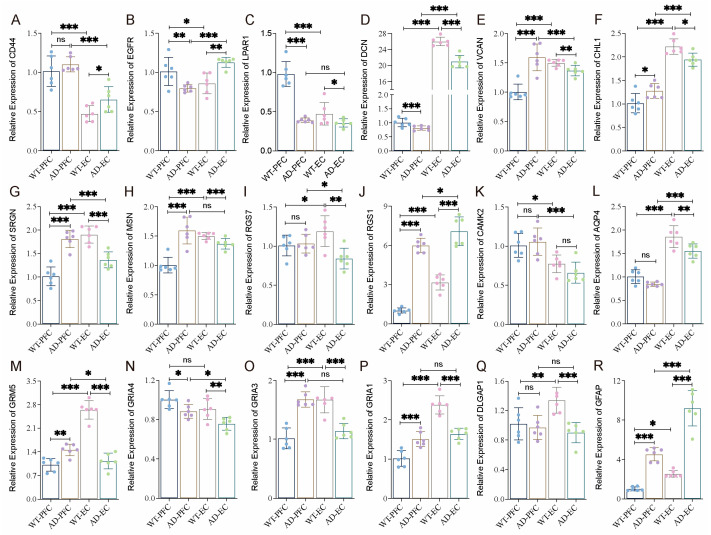
Differential expression of key regulated genes in the PFC and EC of WT and AD mice. (**A**–**R**) The expression levels of 18 hub genes in Figure 6B were determined by RT-qPCR in the PFC and EC of WT and AD mice (three males and three females). Data were presented as mean ± SD, *n* = 6/group. * *p* < 0.05, ** *p* < 0.01, *** *p* < 0.001. ns, no significance.

To validate these results, we selected the generally accepted mouse model of AD for experimental studies due to the difficulty of obtaining human samples. Our previous studies suggested that cognitive impairment (water maze test) and pathological changes (Aβ) began to appear in AD mice at 9–10 months of age. Therefore, 10-month-old mice were selected for subsequent studies. Using quantitative RT-PCR, we systematically examined the cortical expression patterns of the 18 key network genes identified in our human studies. Under normal conditions (WT mice), except for *GRIA4*, 17 of 18 genes showed statistically significant expression differences between PFC and EC (Figure 7). This widespread transcriptional divergence underscores the inherent molecular specialization of different cortical areas, even in healthy states. The expression of these key genes in AD mice varies between the PFC and EC regions (Figure 7A–R). Compared with wild-type mice, *CD44*, *RGS1*, and *GFAP* showed consistent upregulation, but *LPAR1*, *DCN*, *AQP4*, *GRIA4*, and *DLGAP* exhibited consistent downregulation in AD mice across two regions. The other eight genes (*SRGN*, *EGFR*, *VCAN*, *CHL1*, *MSN*, *CAMK2A*, *GRM5*, *GRIA3*, and *GRIA1*) showed opposite expression patterns in EC and PFC (Figure 7). On the whole, the gene expression difference in EC is significantly stronger than that of PFC; expression alterations of *CD44*, *RGS7*, *AQP4*, and *DLGAP1* in EC were significantly more pronounced compared with PFC, indicating that EC plays a more important role in the occurrence and development of AD.

## 3. Discussion

Our integrated snRNA-seq analysis of PFC and EC tissues from AD patients revealed gene dysregulation across major neural cell types, including ExN, InN, OPC, Astro, Micro and Oligo. In addition, we also identified significant transcriptional alterations in Endo cells and a distinct population of Unknown cells. EC exhibited pronounced molecular changes, with approximately 20-fold more DEGs compared with PFC across all cell types. Interactions between the key regulatory genes of the different cell types constitute the cellular regulatory networks associated with AD pathogenesis. These findings were further validated in AD mouse models, where RT-qPCR analysis of 18 key network genes revealed significantly different expression between AD and WT mice, with more prominent gene expression differences observed in EC than PFC regions.

The complexity of neurodegenerative disorders like AD necessitates a detailed understanding of each cell type’s contribution to disease progression. Recent advances in scRNA/snRNA-seq technology have provided possibilities to characterize the cell-type-specific dysregulation in normal and pathological conditioned brain tissues [7,9,12]. In the present study, functional enrichment analyses revealed that DEGs of EC Astro were mainly involved in pathways related to ensheathment of neurons and oligodendrocyte differentiation, while DEGs in EC Oligo participated in learning, memory, and cognition pathways. These suggested crucial Astro–Oligo interactions in EC may potentially underlie cognitive impairment in AD. However, DEGs of PFC Astro Oligo differed significantly from those in the EC cells. PPI analysis identified an interconnected network of Astro key regulators—*VCAN*, *CD44*, *GFAP*, *AQP4*, and *CNTNAP2*. Differential expression of these genes between PFC and EC suggests a region-specific dysregulation of critical astrocytic functions in AD. Among these genes, *AQP4* showed opposite expression alterations in the two brain regions. Previous studies reported the absence of Astro *AQP4* in animal models exhibited a 70% reduction in CSF influx and stromal solute clearance through the glymphatic system, suggesting that most of the liquid flow between the inflow and outflow routes of these anatomical structures was correlated with the expression of Astro *AQP4* [20,21]. *AQP4* mediates the clearance of soluble Aβ and tau in the CNS [20,22,23], and disrupted *AQP4* expression results in rapid accumulation of pathological soluble proteins and abnormal tau [20,22,24,25]. The opposite changes in *AQP4* expression observed across PFC and EC regions in AD patients suggested that the glymphatic system of these two cortical tissues may exhibit different functions in AD patients. Further investigations on the dynamics of specific regions of human CSF by longitudinal imaging studies are promising and will provide a basis for the early diagnosis of AD or related diseases. Simultaneously, region-specific therapeutic strategies targeting *AQP4* may be potentially beneficial for the diagnosis and treatment of AD.

DEGs of EC Micro were prominently involved in glutamatergic synaptic transmission and nervous system development, potentially contributing to neuronal loss and volume reduction in EC of AD patients. Key Micro regulators revealed by the PPI network included *PTPRC*, *MSR1*, *APOE*, *B2M*, and *NRXN3*. Multiple large-scale genome-wide association studies and genome-wide association meta-analyses have demonstrated that the *APOEε4* allele is the strongest genetic risk factor for sporadic AD [26]. *APOE* and *TREM2* risk variants are associated with a significant reduction in *CD163*-positive amyloid-reactive Micro [27]. However, few studies have reported on treatment strategies targeting *APOE*. *B2M* is a component of major histocompatibility complex class 1 (*MHCI*) molecules, which negatively regulates hippocampal function in an age-dependent manner. In a previous study, systemic accumulation of *B2M* in the blood of aging individuals (mice and humans) promotes age-related cognitive dysfunction and impairment of neurogenesis [28]. Our study revealed elevated *B2M* expression in both PFC and EC Micro, suggesting its potential as a therapeutic target for AD research. Antioxidant molecule 1 (*OXR1*) is a protein-coding gene that plays a role in resisting oxidative stress [29]. Individuals with *OXR1* deficiency have severe neurodevelopmental impairments [30]. A transcriptome analysis revealed that OXR1 overexpression in neurons would significantly delay the non-cell-autonomous neuroinflammatory response, activation of the classical complement system, and *STAT3* activation [31]. A separate study pointed out that *OXR1* depletion aggravated neurodegenerative disorders in Parkinson’s disease [32]. These findings position *OXR1* as a promising target for neuroprotective strategies across multiple neurodegenerative diseases.

It is worth pondering that the number of Oligo increased significantly either in PFC or EC of AD patients. Transcriptional regulation showed modest changes, with two genes overlapping—*CTNNA2* and *LINC01099*. In primate brain structures, catenin protein family members are thought to play important roles in the folding and laminarization of the cerebral cortex, which has a unique pattern of neuronal gene expression characterized by high folding and stratification. High levels of these transcripts support the catenin functioning even in the adult brain [33]. In the present study, *CTNNA2* expression was downregulated in PFC/EC Oligo and PFC InN. Whole genome sequencing (WGS) showed an association of *CTNNA2* variation signals with risk in AD patients [34]. CTNNA2 might be closely related to the occurrence and development of AD. *LINC01099* expression was upregulated in PFC Oligo but downregulated in EC Oligo. Little is known about the major function of *LINC01099*. However, genome-wide association studies (GWAS) revealed the association of *LINC01099* with multiple neurodegenerative diseases such as Huntington disease and AD. On this basis, we identified five lncRNA-miRNA-mRNA axes: *LINC01099*/*miR-758-3p*/*RORA*, *LINC0109*9/*miR-146a-5p*/*LRP2*, *LINC01099*/*miR-377-5p*/*NRXN3*, *LINC01099*/*miR-221-3p*/*FOS*, and *LINC01099*/*miR-222-3p*/*FOS*.

AD is often accompanied by disruption of cellular cholesterol balance, and miR-758 has been identified as a potent post-transcriptional regulator of lipid metabolism genes [35]. RORA is upregulated in the AD hippocampus and significantly correlates with AD pathology [36,37,38,39]. Moreover, several genes in the network interacting with RORA, such as *APP*, *DNM1L*, and *TIA1*, are all associated with AD, solidifying the critical role of *RORA* in the pathology/etiology of AD [40]. In this study, the expression of both *LINC01099* and *RORA* was downregulated in EC Oligo, suggesting the potency of the *LINC01099*/*miR-758-3p*/*RORA* axis in AD pathogenesis. *MicroRNA-146a-5p* regulates the inflammatory response, and the functional SNP of *miR-146a* is associated with AD risk [41]. The elevated expression of *miR-146a-5p* in AD patient serum and blood extracellular vesicles was associated with AD severity [42,43,44]. In addition, the low-density lipoprotein receptor-associated protein-2 (*LRP2*) is known to play important roles in AD pathogenesis [45,46,47,48]. These results indicated that the *LINC01099*/*miR-146a-5p*/*LRP2* axis may serve as a key pathway for AD intervention. In the AD population, the prevalence of obstructive sleep apnea (OSA) was five times higher than in cognitively non-impaired individuals, and *miR-377-3p* is significantly elevated in the plasma of AD patients with OSA [49]. GWAS revealed that *NRXN3* was correlated with male AD susceptibility [50,51]. Our integrated data demonstrated the elevated levels of *LINC01099* and *NRXN3* in PFC, suggesting a possible regulation among *LINC01099*, *miR-377-5p*, and *NRXN3* in AD. *MiR-221* may promote the accumulation of Aβ protein in AD brain tissue, overexpression of which in SH-SY5Y causes a decrease in ADAM10 expression, an α-secretase that decomposes the Aβ protein precursor in the AD non-amyloid protein formation pathway [52]. In addition, *miR-222* downregulation was ever found in *APP*swe/*PSΔE9* mice (AD model) [53], in CSF [54], and in serum [55,56] of AD patients. Aberrant expression of *miR-222* may contribute to dysregulation of the AD cell cycle by affecting p27Kip1 expression [53]. Researchers found that disease-associated Micro (DAM) (i.e., *PAK1*, *MAPK14*, and *CSF1R*) and disease-related Astro (DAA) (i.e., *NFKB1*, *FOS*, and *JUN*) were all significantly enriched for neuroinflammatory pathways and known genetic variants [57]. The number of *JUN*- and *FOS*-positive neurons increased in hippocampal regions in almost all AD patient postmortem brain samples [58]. *JUN* and *FOS* immunoreactivity was also associated with *GFAP*-positive Astro that are distributed in the AD cortex and around AD brain thiamin-stained plaques [59]. Members of the *FOS* family play important roles in Aβ and tau accumulation, nerve fiber tangles, and programmed cell death [60]. Thus, *LINC01099* may influence *FOS* expression through two different pathways—*miR-221-3p* and *miR-222-3p*. As described above, *FOS* initiates the inflammatory response in Micro, while in this study, FOS downregulation was observed in PFC and EC Micro but increased in EC Oligo. This implied that Oligo may be involved in the initiation of the inflammatory response in EC Micro. These two pathways, *LINC01099*/*miR-221-3p*/*FOS* (EC_up) and *LINC01099*/*miR-222-3p*/*FOS* (EC_up), might be central to the inflammatory response modulation in Oligo-triggered Micro. Differential expression of *LINC01099* and *FOS* across different brain regions (PFC, EC, and hippocampus) necessitates more detailed elucidation of precise cellular and molecular signatures in distinct brain regions for AD treatment.

Our results revealed consistent expression patterns of overlapped DEGs for PFC and EC OPC, and these DEGs were significantly associated with functions related to glial formation and synaptic transmission. Among these shared DEGs, *NEAT1* and *ST18* have been well documented in AD pathogenesis [61,62,63,64], showing consistent upregulation in AD patients and animal models that promotes the disease progression [62,63,64]. While the roles of *XYLT1*, *RNF220*, *KCTD8*, and *SHISA9* have not been previously linked to AD, their known functions related to nervous system development [65,66,67,68,69,70,71,72], white matter dystrophy [73], affective-related behavior [74], synaptic plasticity [75,76,77,78,79], visual integration [80], and schizophrenia [81] suggest potential roles in AD pathogenesis. This is particularly relevant given the established connections between AD and various conditions, including brain development [82,83], type II diabetes [84,85,86,87], cancer [88], schizophrenia [89,90], and periodontitis [91,92,93,94,95].

Both ExN and InN exhibited parallel degenerative patterns in AD patients, with reductions in cell numbers across the AD PFC and EC regions. However, transcriptomic analysis revealed substantially more DEGs in both neuronal populations of the EC region compared with the PFC. The PPI network uncovered several pivotal DEGs, including *CAMK2A*, *SLC6A1*, *GRIA4*, and *STMN2*. *CAMK2A* dysregulation potentially disrupts the synaptic plasticity mechanisms crucial for memory formation [96,97,98,99]. Equally compelling was the involvement of *SLC6A1*, encoding the *GABA* transporter *GAT-1*, whose altered expression could significantly impact the delicate balance of cortical inhibition [100,101]. The AMPA receptor subunit *GRIA4* [102,103] and microtubule regulator *STMN2* [104,105] completed this network of dysfunction, together painting a picture of compromised excitatory transmission and impaired neuronal maintenance. These dysregulations in the different brain regions of AD patients might be crucially functional in the pathophysiology of AD, and their precise pathogenic mechanisms remain to be fully elucidated. Turning to InN, *GRM5* and *MDGA2* are key nodes revealed by PPI networks. The metabotropic glutamate receptor GRM5 showed opposite alterations in PFC and EC InN. The *GRM5*-involved signaling pathway activates the phosphatidylinositol–calcium second messenger system, and *GRM5* helps maintain neural network activity and synaptic plasticity [106,107,108,109,110,111,112,113,114]. Accumulating evidence demonstrated that *GRM5* is involved in the pathological feature formation of AD and represents a potential target for disease-modifying intervention [115,116]. *MDGA2* is a potential specific factor affecting the presynaptic neurotransmitter recruitment of gliin-2 at inhibitory synapses [117]. Targeted mutations in *MDGA1* and *MDGA2* enhance both inhibitory and excitatory synapses in mouse hippocampal pyramidal neurons, respectively. However, *MDGA2* blocked the interaction of gliin-1 with neuroligin and inhibited the development of excitatory synapses. The relative levels and subcellular distribution of neuroligin and neuroligin determine the synaptic-specific tissue properties of *MDGA* [118,119,120,121]. Differential expression of *GRM5* and *MDGA2* in the PFC and EC suggests that the imbalance degree of excitatory and inhibitory synapses in different brain regions of AD patients may contribute to the diverse clinical features of AD patients. This nuanced understanding of synaptic disruption could prove invaluable for developing targeted therapies that address specific symptom domains in AD.

Beyond classical neuronal populations, Endo and Unknown cells displayed significant alterations across two brain regions in AD patients. Both Endo and Unknown cells in EC embody more DEGs as compared with the PFC. However, their functional contributions to AD pathogenesis appear distinct. Endo cells showed a particularly strong association with synaptic signaling, suggesting an underappreciated role in maintaining neuronal communication. Our PPI network pinpointed *EGFR* as a central regulator in Endo pathology. This finding gains clinical relevance from emerging evidence that *EGFR* inhibitors can ameliorate pathological and behavioral conditions in neurodegenerative diseases, including AD and amyotrophic lateral sclerosis (ALS). While these preliminary findings are promising, the precise mechanisms underlying vascular *EGFR* signaling influencing AD progression present an important avenue for future research. Unknown cells demonstrated transcriptomic profiles linked to nervous system development. The PPI network identified *GRIN2B*, encoding the *NR2B* subunit of *NMDA* receptors, as a key regulatory node in these cells. We observed the upregulation of *GRIN2B* in the PFC and downregulation in the EC of AD patients, aligning with previous reports of elevated *GRIN2B* expression in the frontal regions of MCI and AD mice [101]. Comprehensive analysis of miRNA and mRNA expression profiles of postmortem brain samples from AD patients identified *GRIN2B* as one of the key genes in DEGs modules [122]. GWAS analysis linked *GRIN2B* SNP loci to AD risk [123,124,125]. Although the exact pathogenic role of *GRIN2B* in AD remains ambiguous, our present findings uncovered its involvement in synaptic dysfunction through region-specific expression alterations or genetic association with disease risk. The contrasting regulation of *GRIN2B* between cortical regions may contribute to the differential vulnerability observed in AD, potentially explaining why certain brain areas succumb to pathology earlier than others.

Our study characterized gene expression changes across multiple independent cell types in AD-affected PFC and EC. To contextualize these findings within established AD molecular subtypes, we performed a comparative analysis with the results of Neff RA et al. [126], which grouped 155 AD participants into three major classes (A, B, and C) and five subtypes annotated as A, B1, B2, C1, and C2. In general, our molecular characteristics were strongly consistent with Neff’s broad classifications (A, B, and C). The similarity between PFC/EC DEGs (our work) and DEGs in AD subtypes (Neff) was smaller when analyzed with finer subtypes (A, B1, B2, C1, C2) (Figure S5A). When analyzed with broad subtypes (A, B, C), a greater transcriptomic similarity was observed between our work and Neff’s study (Figure S5B). However, this concordance was not shared by each individual AD subtype, and opposite trends were found in PFC and EC (Figure S5B). Gene expression changes in the PFC showed consistency with class A and B subtypes, whereas the EC alterations mirrored class C subtypes (Figure S4B). Functionally, both EC and class C profiles showed significant enrichment for Aβ-binding activity.

Our outcomes unveiled complex cell networks involved in neurotransmitter activity, ion transport, synaptic plasticity, Aβ protein binding. and other AD-related essential pathways. Crucial AD-associated genes were identified in each cell type, including *RGS1*, *GRIA1*, *GRIA3*, *GRIA4*, *DLGAP1*, *MSN*, *CHL1*, *DCN*, *LPAR1*, *EGFR*, *GRM5*, *CAMK2A*, *APOE*, *AQP4*, *GFAP*, and *CD44*, showing potential involvement in AD. Importantly, cross-model validation using human AD organoids demonstrated conserved dysregulation of several key genes (*CD44*, *CHL1*, *MSN*, *RGS7*, *GRIA1*, *CAMK2A*, *DLGAP1*, *GFAP*) between postmortem brain tissue and organoid models, while also revealing model-specific differences that highlight both the utility and limitations of current in vitro systems for AD research. The dysregulation of this cell regulatory network varies in different brain regions in AD patients, and the generalizability of these data will depend on independent validation of other AD cohort studies. While our multi-model approach (incorporating human snRNA-seq, APP/PS1 mice, and organoid data) strengthens the biological relevance of these findings, we acknowledge several limitations: (1) The APP/PS1 model primarily recapitulates amyloid pathology without full tauopathy or neurodegeneration; (2) organoid models, while valuable for studying human-specific pathways, lack the complete cellular diversity and architectural organization of native brain tissue; and (3) the generalizability of our regional findings requires validation in additional cohorts with different genetic backgrounds and disease stages. Our study also has limitations regarding sex balance. The predominance of male samples in our human snRNA-seq analysis may affect generalizability, given established sex differences in AD risk and progression. While our mouse validation experiments used balanced sex groups, the small sample sizes limited our ability to thoroughly investigate sex-specific effects. Future studies with larger, sex-balanced cohorts will be important to determine whether the cellular networks we identified show sex-dependent regulation.

Nonetheless, this work provides a cellular-resolution framework for understanding AD heterogeneity. Differential dysregulation of cellular regulatory networks across the PFC and EC allows investigating brain region-specific precision medicine therapies and cell-type targeted interventions, such as repetitive transcranial magnetic stimulation (rTMS), transcranial direct current stimulation (tDCS) [127], and selective endogenous encapsidation for cellular delivery (SEND) RNA delivery technology [128]. While the generalizability of these findings requires validation in additional cohorts, our work establishes an important paradigm for understanding AD as a disorder of cellular networks, with distinct pathological processes unfolding in different brain regions. The regional specificity of cellular dysregulation patterns uncovered in our study suggests that future therapeutic strategies may need to account for both cell type and anatomical location to achieve optimal efficacy. Importantly, our findings bridge the gap between bulk tissue analyses and cellular pathophysiology, providing a robust foundation for developing next-generation AD treatments that move beyond one-size-fits-all approaches.

## 4. Materials and Methods

### 4.1. Datasets Collection

SnRNA-seq datasets integrated in this study (GSE141552, GSE157827, and GSE138852) were downloaded from Gene Expression Omnibus (GEO) of National Center for Biotechnology Information (https://www.ncbi.nlm.nih.gov/geo/) (accessed on 10 June 2021). GSE141552 and GSE157827 were obtained using the 10× Genomics and NovaSeq 6000 sequencing platform (Illumina, Inc., San Diego, CA, USA), and GSE138852 was generated using the 10× Genomics and NextSeq 500 sequencing platform (Illumina, Inc., San Diego, CA, USA) [7,9,12]. The integrated 3 snRNA-seq datasets comprised 8 PFC_AD, 10 PFC_Ct, 6 EC_AD, and 6 EC_Ct samples (Table 1 and Table S1). The scRNA-seq data of brain organoids used in this study are available under accession number GSE164089 [19], which was generated with 10× Genomics and NovaSeq 6000 sequencing platform.

### 4.2. SnRNA-Seq Data Analysis

#### 4.2.1. Preprocessing, Quality Control, and Data Integration

The gene barcode matrices for each sample were loaded into R software v.4.2.2 using the Read 10× function in the Seurat R package v.4.2.0 [129]. The Seurat Object, corresponding to each sample, was created using the *CreateSeuratObject* function with the input gene barcode matrix provided as the raw data. The datasets were integrated using the method of Stuart et al. [13]. Data quality was strictly controlled prior to integration. Nuclei with ≤200 genes, ≥2500 unique molecular identifiers, or ≥5% mitochondrial genes were filtered out to exclude potential dead cells and cell debris. In total, 124,658 high-quality nuclei were obtained for subsequent analyses. For the integration analysis, the highly variable features of each sample were identified using the *FindVariableFeatures* function, with parameter set as *selection.method* = vst, *nfeatures* = 2000. The features of the samples were anchored using the *FindIntegrationAnchors* function with the parameter *dims* = 1:20. All samples were integrated using the *IntegrateData* function with the parameter *dims* = 1:20.

#### 4.2.2. Data Dimension Reduction and Clustering Analysis

We scaled the expression matrix and performed a linear dimension reduction using the *RunPCA* function with the parameter *npcs* = 50. The *p*-value distribution of each major component was visualized using the *JackStrawPlot* function and selected to perform graph-based clustering using the first 30 principal components. K-nearest neighbor (KNN) clustering was followed using the *FindClusters* function with the parameter *resolution* = 1, and UMAP clustering visualized a total of 31 cell clusters using the *RunUMAP* function with the parameter *dims* = 1:30. Wilcoxon rank-sum test was performed to identify DEGs in each cell cluster using the *FindAllMarkers* function with the parameters *logfc.threshold* = 0.25 and *test.use* = Wilcox. Then, a cell-type identity was assigned to each cell cluster based on expression patterns of known cell-type markers and additional cell-type-specific marker genes. For cell-type markers, the level of statistical significance was set at an adjusted *p*-value < 0.1.

#### 4.2.3. Examination of Cell Type-Specific Transcriptomic Changes

To examine the cell-type-specific transcriptomic changes in AD, we compared the transcriptome profiles of individual cell types among PFC_AD samples, EC_AD samples, PFC_Ct, and EC_Ct by the Wilcoxon rank-sum test using the *FindMarkers* function with the parameters *logfc.threshold* = 0.25 and *test.use* = Wilcox.

### 4.3. Gene Ontology (GO) and Kyoto Encyclopedia of Genomes and Genes (KEGG) Signaling Pathway Enrichment Analysis

In this study, DEGs were all mapped to the GO terms in the GO database (https://geneontology.org/) (accessed on 18 July 2021), and the number of genes for each term was calculated. Pathway-based analysis was used to characterize the biological functions of the genes. Pathway enrichment analysis identified significant signal transduction pathways in the KEGG database (http://www.genome.jp/kegg/) (accessed on 18 July 2021). R software v.4.2.2 (http://www.r-project.org) (accessed on 10 July 2021) and multiple R packages, such as *clusterProfiler*, *org.Hs.eg.db*, *enrichplot,* and *ggplot2*, were used to map the bars, bubble maps, and signaling pathway maps of GO and KEGG enrichment analysis.

### 4.4. Protein-Protein Interactions (PPI) Network Analysis

The STRING database (https://www.string-db.org/) (accessed on 20 July 2021) was used for DGE-associated protein interaction analysis and to construct a PPI network. Cytoscape 3.8.0 was used (https://cytoscape.org/) (accessed on 20 July 2021) to construct the cell differential expression network.

### 4.5. Animal Care and Grouping

Ten-month-old APP/PS1 transgenic mice (AD mice, *n* = 6, 3 males and 3 females) and wild-type mice (WT mice, *n* = 6, 3 males and 3 females) of C57BL/6 strain were provided by the Experimental Animal Center of Kunming Medical University. Animals were kept under standard conditions in the SPF laboratory. This study was approved by Animal Care and Use Committee of Kunming Medical University (kmmu2019058). All experimental procedures, including animal care and testing, were conducted in accordance with the United States Public Health Service’s Policy on Humane Care and Use of Laboratory Animals. All mice were deeply anesthetized before euthanasia and were immediately perfused with precooled 0.9% normal saline until the liver turned white. Their brains were harvested, with PFC and EC regions collected for the following experiment.

### 4.6. Real-Time Quantitative Polymerase Chain Reaction (RT-qPCR)

The total RNA fraction was extracted using Trizol reagent (Takara Bio Inc., Otsu, Japan) and reverse transcribed into complementary DNA (cDNA) using RevertAid First Strand cDNA Synthesis System (Invitrogen, Thermo Fisher Scientific, Waltham, MA, USA). Quantitative PCR reactions were carried out with Power SYBR (DBI Bioscience, Shanghai, China) according to the manufacturer’s instructions. The expression level of targeted gene was normalized to that of GAPDH using the 2^−ΔΔct^ method. Primers used for the reactions are shown in Table 2.

### 4.7. Data Visualization and Statistical Analysis

The sequencing data were analyzed using R software v.4.2.2 and Cell Ranger v.6.0.1. All data are presented as the primary data or mean ± SEM. Statistical analyses for RT-qPCR were performed using SPSS 19.0 software. One-way analysis of variance (ANOVA) with Tukey’s post hoc test was applied for comparison among multiple groups. The data were visualized using Seurat’s *DoHeatmap*, *DotPlot* function, TBtools v.1.098, and Cytoscape v.3.8.0) where appropriate. GraphPad Prism software v.7.0 (GraphPad Software Inc., San Diego, CA, USA) was used for quantification and histogram generation. *p* value < 0.05 was considered to be statistically significant.

## 5. Conclusions

Through integrated analysis of snRNA-seq data from the PFC and EC of AD patients, we systematically uncovered the cellular network dysregulation underlying disease pathogenesis. Our findings revealed profound transcriptional disturbances across major neural cell types, including Oligo, ExN, InN, OPC, Astro, and Micro, as well as in non-neural populations such as Endo and a distinct cluster of uncharacterized cells. Notably, the EC emerges as the molecular epicenter of early AD pathology, exhibiting a much greater burden of DEGs compared with the PFC. Our reconstructed cross-cellular interaction network identifies key regulatory genes forming multidimensional regulatory axes to drive AD pathogenesis. RT-qPCR validation not only recapitulated the expression patterns of 18 core genes identified in human samples but further demonstrated substantially greater expression divergence in EC versus PFC. These findings suggest that neurovascular units and enigmatic cell clusters may cooperatively participate in tau hyperphosphorylation and Aβ deposition through specialized signaling cascades. This study fundamentally reshapes our understanding of AD as a disorder of integrated cellular networks, providing transformative insights into cell type-specific vulnerability patterns and opening new avenues for targeted therapeutic development against AD.

## Figures and Tables

**Table 1:** Sample information.

Stage	Age (Years)	Sex	Datasets	Ref.
PFC_Ct	56	M	GSE141552	[12]
	56	M	GSE141552	[12]
	69	M	GSE141552	[12]
	74	F	GSE157827	[9]
	78	M	GSE157827	[9]
	79	F	GSE157827	[9]
	85	M	GSE157827	[9]
	90	M	GSE157827	[9]
	93	M	GSE157827	[9]
	94	F	GSE157827	[9]
PFC_AD	60	F	GSE157827	[9]
	63	M	GSE157827	[9]
	69	M	GSE157827	[9]
	71	M	GSE157827	[9]
	72	M	GSE157827	[9]
	84	M	GSE157827	[9]
	85	M	GSE157827	[9]
	95	M	GSE157827	[9]
EC_CT	67.3	F	GSE138852	[7]
	72.6	M	GSE138852	[7]
	75.6	M	GSE138852	[7]
	77.5	M	GSE138852	[7]
	82.7	F	GSE138852	[7]
	82.7	M	GSE138852	[7]
EC_AD	67.8	F	GSE138852	[7]
	73	M	GSE138852	[7]
	74.6	M	GSE138852	[7]
	83	F	GSE138852	[7]
	83.8	M	GSE138852	[7]
	91	M	GSE138852	[7]

**Table 2:** Primer sequences of detected genes.

Genes	Primers	Sequences
*VCAN*	Forward primer	GGTGTCACAACCCGCATTTG
	Reverse primer	TAACAGGTGGGCTGGTTTCC
*DCN*	Forward primer	GTGCTATGGAGTAGAAGCAGGA
	Reverse primer	ACACTGCACCACTCGAAGAT
*EGFR*	Forward primer	AGCTGGCATCATGGGAGAGA
	Reverse primer	CTGCCATTGAACGTACCCAGA
*CD44*	Forward primer	CACCTTGGCCACCACTCCTAAT
	Reverse primer	TGACTTGGATGGTTGTTGTGGG
*CHL1*	Forward primer	GGATCTCTGTGGGCAGATCG
	Reverse primer	GAGGCAACGTGCAAAGACTG
*SRGN*	Forward primer	CCAGGCAGGTCAGAGGAAACTG
	Reverse primer	AAGCCATTCGGTTTGCAGCG
*MSN*	Forward primer	TGAGAACATGCGACTGGGAC
	Reverse primer	GGCTCCAGCACAGTGTTAGT
*LPAR1*	Forward primer	CCTTTGGCCAGGCTTACAGTT
	Reverse primer	GCCAACATGATGAACACGCA
*RGS1*	Forward primer	CCATCTCCATGCCAAGGTTGA
	Reverse primer	CATTTTGACCTGTCTGGTTGGC
*RGS7*	Forward primer	CTCCGGGTCAGACATTGTTCA
	Reverse primer	TGAAACCGGTAGAAGGTGCC
*GRIA1*	Forward primer	ATGTGGAAGCAAGGACTCCG
	Reverse primer	GGATTGCATGGACTTGGGGA
*GRIA3*	Forward primer	CGAGAGCAAGTTGAGGGGAG
	Reverse primer	CTGTGCTCCTGTACCGTGTT
*GRIA4*	Forward primer	AAGGCTATGGTGTAGCGACG
	Reverse primer	TCAAGGCACTCGTCTTGTCC
*CAMK2A*	Forward primer	CTGACCATCAACCCGTCCAA
	Reverse primer	CAGAAGATTCCTTCACACCATCG
*GRM5*	Forward primer	TTGCCTGCTTCTCAGTTGTCT
	Reverse primer	CTCAGGAAGCACCACTAGGAC
*DLGAP1*	Forward primer	CATCTGCTCTGGGACTTGCT
	Reverse primer	GCTTGTGGTCTGAGTGGTGT
*AQP4*	Forward primer	GACAAGTGCCCGTAATCTGAC
	Reverse primer	ACAGTCACAGCGGGATTGAT
*GFAP*	Forward primer	AAGCTCCAAGATGAAACCAAC
	Reverse primer	TTCTCTCCAAATCCACACGA

## Data Availability

All snRNA-seq data were obtained from the Gene Expression Omnibus (GEO) public repository: accession numbers GSE141552, GSE157827, and GSE138852 (https://www.ncbi.nlm.nih.gov/geo/) (accessed on 10 June 2021). The corresponding data used to support the findings of this study are included within the Supplementary Materials.

## Supplementary Materials

The following supporting information can be downloaded at: https://www.mdpi.com/article/10.3390/ijms26104841/s1.

## Abbreviations

The following abbreviations are used in this manuscript:

Oligo	oligodendrocytes
ExN	excitatory neurons
OPC	oligodendrocyte precursor cells
Astro	astrocytes
InN	inhibitory neurons
Endo	endothelial cells
Micro	microglia
AD	Alzheimer’s disease

## References

[1] Kunkle B.W., Grenier-Boley B., Sims R., Bis J.C., Damotte V., Naj A.C., Boland A., Vronskaya M., van der Lee S.J., Amlie-Wolf A. (2019). Genetic meta-analysis of diagnosed Alzheimer’s disease identifies new risk loci and implicates Aβ, tau, immunity and lipid processing. Nat. Genet..

[2] Wingo A.P., Liu Y., Gerasimov E.S., Gockley J., Logsdon B.A., Duong D.M., Dammer E.B., Robins C., Beach T.G., Reiman E.M. (2021). Integrating human brain proteomes with genome-wide association data implicates new proteins in Alzheimer’s disease pathogenesis. Nat. Genet..

[3] Sierksma A., Lu A., Mancuso R., Fattorelli N., Thrupp N., Salta E., Zoco J., Blum D., Buée L., De Strooper B. (2020). Novel Alzheimer risk genes determine the microglia response to amyloid-β but not to TAU pathology. EMBO Mol. Med..

[4] Habib N., McCabe C., Medina S., Varshavsky M., Kitsberg D., Dvir-Szternfeld R., Green G., Dionne D., Nguyen L., Marshall J.L. (2020). Disease-associated astrocytes in Alzheimer’s disease and aging. Nat. Neurosci..

[5] Keren-Shaul H., Spinrad A., Weiner A., Matcovitch-Natan O., Dvir-Szternfeld R., Ulland T.K., David E., Baruch K., Lara-Astaiso D., Toth B. (2017). A Unique Microglia Type Associated with Restricting Development of Alzheimer’s Disease. Cell.

[6] Chen W.T., Lu A., Craessaerts K., Pavie B., Sala Frigerio C., Corthout N., Qian X., Laláková J., Kühnemund M., Voytyuk I. (2020). Spatial Transcriptomics and In Situ Sequencing to Study Alzheimer’s Disease. Cell.

[7] Grubman A., Chew G., Ouyang J.F., Sun G., Choo X.Y., McLean C., Simmons R.K., Buckberry S., Vargas-Landin D.B., Poppe D. (2019). A single-cell atlas of entorhinal cortex from individuals with Alzheimer’s disease reveals cell-type-specific gene expression regulation. Nat. Neurosci..

[8] Mathys H., Davila-Velderrain J., Peng Z., Gao F., Mohammadi S., Young J.Z., Menon M., He L., Abdurrob F., Jiang X. (2019). Single-cell transcriptomic analysis of Alzheimer’s disease. Nature.

[9] Lau S.F., Cao H., Fu A.K.Y., Ip N.Y. (2020). Single-nucleus transcriptome analysis reveals dysregulation of angiogenic endothelial cells and neuroprotective glia in Alzheimer’s disease. Proc. Natl. Acad. Sci. USA.

[10] Berchtold N.C., Coleman P.D., Cribbs D.H., Rogers J., Gillen D.L., Cotman C.W. (2013). Synaptic genes are extensively downregulated across multiple brain regions in normal human aging and Alzheimer’s disease. Neurobiol. Aging.

[11] Wang M., Roussos P., McKenzie A., Zhou X., Kajiwara Y., Brennand K.J., De Luca G.C., Crary J.F., Casaccia P., Buxbaum J.D. (2016). Integrative network analysis of nineteen brain regions identifies molecular signatures and networks underlying selective regional vulnerability to Alzheimer’s disease. Genome Med..

[12] Brenner E., Tiwari G.R., Kapoor M., Liu Y., Brock A., Mayfield R.D. (2020). Single cell transcriptome profiling of the human alcohol-dependent brain. Hum. Mol. Genet..

[13] Stuart T., Butler A., Hoffman P., Hafemeister C., Papalexi E., Mauck W.M., Hao Y., Stoeckius M., Smibert P., Satija R. (2019). Comprehensive Integration of Single-Cell Data. Cell.

[14] Miao Y.R., Liu W., Zhang Q., Guo A.Y. (2018). lncRNASNP2: An updated database of functional SNPs and mutations in human and mouse lncRNAs. Nucleic Acids Res..

[15] Vlachos I.S., Zagganas K., Paraskevopoulou M.D., Georgakilas G., Karagkouni D., Vergoulis T., Dalamagas T., Hatzigeorgiou A.G. (2015). DIANA-miRPath v3.0: Deciphering microRNA function with experimental support. Nucleic Acids Res..

[16] Chen Y., Wang X. (2020). miRDB: An online database for prediction of functional microRNA targets. Nucleic Acids Res..

[17] Huang H.Y., Lin Y.C., Li J., Huang K.Y., Shrestha S., Hong H.C., Tang Y., Chen Y.G., Jin C.N., Yu Y. (2020). miRTarBase 2020: Updates to the experimentally validated microRNA-target interaction database. Nucleic Acids Res..

[18] Agarwal V., Bell G.W., Nam J.W., Bartel D.P. (2015). Predicting effective microRNA target sites in mammalian mRNAs. eLife.

[19] Chen X., Sun G., Tian E., Zhang M., Davtyan H., Beach T.G., Reiman E.M., Blurton-Jones M., Holtzman D.M., Shi Y. (2021). Modeling Sporadic Alzheimer’s Disease in Human Brain Organoids under Serum Exposure. Adv. Sci..

[20] Iliff J.J., Wang M., Liao Y., Plogg B.A., Peng W., Gundersen G.A., Benveniste H., Vates G.E., Deane R., Goldman S.A. (2012). A paravascular pathway facilitates CSF flow through the brain parenchyma and the clearance of interstitial solutes, including amyloid β. Sci. Transl. Med..

[21] Rasmussen M.K., Mestre H., Nedergaard M. (2018). The glymphatic pathway in neurological disorders. Lancet Neurol..

[22] Xu Z., Xiao N., Chen Y., Huang H., Marshall C., Gao J., Cai Z., Wu T., Hu G., Xiao M. (2015). Deletion of aquaporin-4 in APP/PS1 mice exacerbates brain Aβ accumulation and memory deficits. Mol. Neurodegener..

[23] Wu J., Carlock C., Shim J., Moreno-Gonzalez I., Glass W., Ross A., Barichello T., Quevedo J., Lou Y. (2021). Requirement of brain interleukin33 for aquaporin4 expression in astrocytes and glymphatic drainage of abnormal tau. Mol. Psychiatry.

[24] Harrison I.F., Ismail O., Machhada A., Colgan N., Ohene Y., Nahavandi P., Ahmed Z., Fisher A., Meftah S., Murray T.K. (2020). Impaired glymphatic function and clearance of tau in an Alzheimer’s disease model. Brain.

[25] Reeves B.C., Karimy J.K., Kundishora A.J., Mestre H., Cerci H.M., Matouk C., Alper S.L., Lundgaard I., Nedergaard M., Kahle K.T. (2020). Glymphatic System Impairment in Alzheimer’s Disease and Idiopathic Normal Pressure Hydrocephalus. Trends. Mol. Med..

[26] Serrano-Pozo A., Das S., Hyman B.T. (2021). APOE and Alzheimer’s disease: Advances in genetics, pathophysiology, and therapeutic approaches. Lancet Neurol..

[27] Nguyen A.T., Wang K., Hu G., Wang X., Miao Z., Azevedo J.A., Suh E., Van Deerlin V.M., Choi D., Roeder K. (2020). APOE and TREM2 regulate amyloid-responsive microglia in Alzheimer’s disease. Acta. Neuropathol..

[28] Smith L.K., He Y., Park J.S., Bieri G., Snethlage C.E., Lin K., Gontier G., Wabl R., Plambeck K.E., Udeochu J. (2015). β2-microglobulin is a systemic pro-aging factor that impairs cognitive function and neurogenesis. Nat. Med..

[29] Durand M., Kolpak A., Farrell T., Elliott N.A., Shao W., Brown M., Volkert M.R. (2007). The OXR domain defines a conserved family of eukaryotic oxidation resistance proteins. BMC Cell Biol..

[30] Wang J., Rousseau J., Kim E., Ehresmann S., Cheng Y.T., Duraine L., Zuo Z., Park Y.J., Li-Kroeger D., Bi W. (2019). Loss of Oxidation Resistance 1, OXR1, Is Associated with an Autosomal-Recessive Neurological Disease with Cerebellar Atrophy and Lysosomal Dysfunction. Am. J. Hum. Genet..

[31] Liu K.X., Edwards B., Lee S., Finelli M.J., Davies B., Davies K.E., Oliver P.L. (2015). Neuron-specific antioxidant OXR1 extends survival of a mouse model of amyotrophic lateral sclerosis. Brain.

[32] Volkert M.R., Crowley D.J. (2020). Preventing Neurodegeneration by Controlling Oxidative Stress: The Role of OXR1. Front. Neurosci..

[33] Smith A., Bourdeau I., Wang J., Bondy C.A. (2005). Expression of Catenin family members CTNNA1, CTNNA2, CTNNB1 and JUP in the primate prefrontal cortex and hippocampus. Brain Res. Mol. Brain Res..

[34] Prokopenko D., Morgan S.L., Mullin K., Hofmann O., Chapman B., Kirchner R., Amberkar S., Wohlers I., Lange C., Hide W. (2021). Whole-genome sequencing reveals new Alzheimer’s disease-associated rare variants in loci related to synaptic function and neuronal development. Alzheimer’s Dement..

[35] Rotllan N., Fernández-Hernando C. (2012). MicroRNA Regulation of Cholesterol Metabolism. Cholesterol.

[36] Porcelli S., Calabrò M., Crisafulli C., Politis A., Liappas I., Albani D., Raimondi I., Forloni G., Benedetti F., Papadimitriou G.N. (2019). Alzheimer’s Disease and Neurotransmission Gene Variants: Focus on Their Effects on Psychiatric Comorbidities and Inflammatory Parameters. Neuropsychobiology.

[37] Baker E., Sims R., Leonenko G., Frizzati A., Harwood J.C., Grozeva D., Morgan K., Passmore P., Holmes C., Powell J. (2019). Gene-based analysis in HRC imputed genome wide association data identifies three novel genes for Alzheimer’s disease. PLoS ONE.

[38] Kochunov P., Glahn D.C., Nichols T.E., Winkler A.M., Hong E.L., Holcomb H.H., Stein J.L., Thompson P.M., Curran J.E., Carless M.A. (2011). Genetic analysis of cortical thickness and fractional anisotropy of water diffusion in the brain. Front. Neurosci..

[39] Hu H., Li H., Li J., Yu J., Tan L. (2018). Genome-wide association study identified ATP6V1H locus influencing cerebrospinal fluid BACE activity. BMC Med. Genet..

[40] Acquaah-Mensah G.K., Agu N., Khan T., Gardner A. (2015). A regulatory role for the insulin- and BDNF-linked RORA in the hippocampus: Implications for Alzheimer’s disease. J. Alzheimer’s Dis..

[41] Zhang B., Wang A., Xia C., Lin Q., Chen C. (2015). A single nucleotide polymorphism in primary-microRNA-146a reduces the expression of mature microRNA-146a in patients with Alzheimer’s disease and is associated with the pathogenesis of Alzheimer’s disease. Mol. Med. Rep..

[42] Wu Y., Xu J., Xu J., Cheng J., Jiao D., Zhou C., Dai Y., Chen Q. (2017). Lower Serum Levels of miR-29c-3p and miR-19b-3p as Biomarkers for Alzheimer’s Disease. Tohoku J. Exp. Med..

[43] Lei B., Liu J., Yao Z., Xiao Y., Zhang X., Zhang Y., Xu J. (2021). NF-κB-Induced Upregulation of miR-146a-5p Promoted Hippocampal Neuronal Oxidative Stress and Pyroptosis via TIGAR in a Model of Alzheimer’s Disease. Front. Cell. Neurosci..

[44] Aharon A., Spector P., Ahmad R.S., Horrany N., Sabbach A., Brenner B., Aharon-Peretz J. (2020). Extracellular Vesicles of Alzheimer’s Disease Patients as a Biomarker for Disease Progression. Mol. Neurobiol..

[45] Qi X.M., Wang C., Chu X.K., Li G., Ma J.F. (2018). Intraventricular infusion of clusterin ameliorated cognition and pathology in Tg6799 model of Alzheimer’s disease. BMC Neurosci..

[46] Spuch C., Ortolano S., Navarro C. (2012). LRP-1 and LRP-2 receptors function in the membrane neuron. Trafficking mechanisms and proteolytic processing in Alzheimer’s disease. Front. Physiol..

[47] Wang L.L., Pan X.L., Wang Y., Tang H.D., Deng Y.L., Ren R.J., Xu W., Ma J.F., Wang G., Chen S.D. (2011). A single nucleotide polymorphism in LRP2 is associated with susceptibility to Alzheimer’s disease in the Chinese population. Clin. Chim. Acta.

[48] Parcerisas A., Rubio S.E., Muhaisen A., Gómez-Ramos A., Pujadas L., Puiggros M., Rossi D., Ureña J., Burgaya F., Pascual M. (2014). Somatic signature of brain-specific single nucleotide variations in sporadic Alzheimer’s disease. J. Alzheimer’s Dis..

[49] Targa A., Dakterzada F., Benítez I.D., de Gonzalo-Calvo D., Moncusí-Moix A., López R., Pujol M., Arias A., de Batlle J., Sánchez-de-la-Torre M. (2020). Circulating MicroRNA Profile Associated with Obstructive Sleep Apnea in Alzheimer’s Disease. Mol. Neurobiol..

[50] Martinez-Mir A., González-Pérez A., Gayán J., Antúnez C., Marín J., Boada M., Lopez-Arrieta J.M., Fernández E., Ramírez-Lorca R., Sáez M.E. (2013). Genetic study of neurexin and neuroligin genes in Alzheimer’s disease. J. Alzheimer’s Dis..

[51] Kohannim O., Hibar D.P., Stein J.L., Jahanshad N., Hua X., Rajagopalan P., Toga A.W., Jack C.R., Weiner M.W., de Zubicaray G.I. (2012). Discovery and Replication of Gene Influences on Brain Structure Using LASSO Regression. Front. Neurosci..

[52] Manzine P.R., Pelucchi S., Horst M.A., Vale F.A.C., Pavarini S.C.I., Audano M., Mitro N., Di Luca M., Marcello E., Cominetti M.R. (2018). microRNA 221 Targets ADAM10 mRNA and is Downregulated in Alzheimer’s Disease. J. Alzheimer’s Dis..

[53] Wang X., Xu Y., Zhu H., Ma C., Dai X., Qin C. (2015). Downregulated microRNA-222 is correlated with increased p27Kip¹ expression in a double transgenic mouse model of Alzheimer’s disease. Mol. Med. Rep..

[54] Dangla-Valls A., Molinuevo J.L., Altirriba J., Sánchez-Valle R., Alcolea D., Fortea J., Rami L., Balasa M., Muñoz-García C., Ezquerra M. (2017). CSF microRNA Profiling in Alzheimer’s Disease: A Screening and Validation Study. Mol. Neurobiol..

[55] Barbagallo C., Mostile G., Baglieri G., Giunta F., Luca A., Raciti L., Zappia M., Purrello M., Ragusa M., Nicoletti A. (2020). Specific Signatures of Serum miRNAs as Potential Biomarkers to Discriminate Clinically Similar Neurodegenerative and Vascular-Related Diseases. Cell. Mol. Neurobiol..

[56] Zeng Q., Zou L., Qian L., Zhou F., Nie H., Yu S., Jiang J., Zhuang A., Wang C., Zhang H. (2017). Expression of microRNA-222 in serum of patients with Alzheimer’s disease. Mol. Med. Rep..

[57] Xu J., Zhang P., Huang Y., Zhou Y., Hou Y., Bekris L.M., Lathia J., Chiang C.W., Li L., Pieper A.A. (2021). Multimodal single-cell/nucleus RNA sequencing data analysis uncovers molecular networks between disease-associated microglia and astrocytes with implications for drug repurposing in Alzheimer’s disease. Genome Res..

[58] Marcus D.L., Strafaci J.A., Miller D.C., Masia S., Thomas C.G., Rosman J., Hussain S., Freedman M.L. (1998). Quantitative neuronal c-fos and c-jun expression in Alzheimer’s disease. Neurobiol. Aging.

[59] Anderson A.J., Cummings B.J., Cotman C.W. (1994). Increased immunoreactivity for Jun- and Fos-related proteins in Alzheimer’s disease: Association with pathology. Exp. Neurol..

[60] Li H., Zhang X., Chen M., Chen J., Gao T., Yao S. (2018). Dexmedetomidine inhibits inflammation in microglia cells under stimulation of LPS and ATP by c-Fos/NLRP3/caspase-1 cascades. Excli. J..

[61] Kim B.H., Nho K., Lee J.M. (2021). Genome-wide association study identifies susceptibility loci of brain atrophy to NFIA and ST18 in Alzheimer’s disease. Neurobiol. Aging.

[62] Zhao M.Y., Wang G.Q., Wang N.N., Yu Q.Y., Liu R.L., Shi W.Q. (2019). The long-non-coding RNA NEAT1 is a novel target for Alzheimer’s disease progression via miR-124/BACE1 axis. Neurol. Res..

[63] Ke S., Yang Z., Yang F., Wang X., Tan J., Liao B. (2019). Long Noncoding RNA NEAT1 Aggravates Aβ-Induced Neuronal Damage by Targeting miR-107 in Alzheimer’s Disease. Yonsei Med. J..

[64] Huang Z., Zhao J., Wang W., Zhou J., Zhang J. (2020). Depletion of LncRNA NEAT1 Rescues Mitochondrial Dysfunction Through NEDD4L-Dependent PINK1 Degradation in Animal Models of Alzheimer’s Disease. Front. Cell. Neurosci..

[65] Song N.N., Ma P., Zhang Q., Zhang L., Wang H., Zhang L., Zhu L., He C.H., Mao B., Ding Y.Q. (2020). Rnf220/Zc4h2-mediated monoubiquitylation of Phox2 is required for noradrenergic neuron development. Development.

[66] Kim J., Choi T.I., Park S., Kim M.H., Kim C.H., Lee S. (2018). Rnf220 cooperates with Zc4h2 to specify spinal progenitor domains. Development.

[67] Zhang L., Ye M., Zhu L., Cha J., Li C., Yao Y.G., Mao B. (2020). Loss of ZC4H2 and RNF220 Inhibits Neural Stem Cell Proliferation and Promotes Neuronal Differentiation. Cells.

[68] Ma P., Song N.N., Cheng X., Zhu L., Zhang Q., Zhang L.L., Yang X., Wang H., Kong Q., Shi D. (2020). ZC4H2 stabilizes RNF220 to pattern ventral spinal cord through modulating Shh/Gli signaling. J. Mol. Cell Biol..

[69] Ma P., Song N.N., Li Y., Zhang Q., Zhang L., Zhang L., Kong Q., Ma L., Yang X., Ren B. (2019). Fine-Tuning of Shh/Gli Signaling Gradient by Non-proteolytic Ubiquitination during Neural Patterning. Cell Rep..

[70] Monteiro R.S., Gentsch G.E., Smith J.C. (2018). Transcriptomics of dorso-ventral axis determination in Xenopus tropicalis. Dev. Biol..

[71] Paus T., Bernard M., Chakravarty M.M., Davey Smith G., Gillis J., Lourdusamy A., Melka M.G., Leonard G., Pavlidis P., Perron M. (2012). KCTD8 gene and brain growth in adverse intrauterine environment: A genome-wide association study. Cereb. Cortex.

[72] Metz M., Gassmann M., Fakler B., Schaeren-Wiemers N., Bettler B. (2011). Distribution of the auxiliary GABAB receptor subunits KCTD8, 12, 12b, and 16 in the mouse brain. J. Comp. Neurol..

[73] Sferra A., Fortugno P., Motta M., Aiello C., Petrini S., Ciolfi A., Cipressa F., Moroni I., Leuzzi V., Pieroni L. (2021). Biallelic mutations in RNF220 cause laminopathies featuring leukodystrophy, ataxia and deafness. Brain.

[74] Bhandari P., Vandael D., Fernández-Fernández D., Fritzius T., Kleindienst D., Önal C., Montanaro J., Gassmann M., Jonas P., Kulik A. (2021). GABA(B) receptor auxiliary subunits modulate Cav2.3-mediated release from medial habenula terminals. eLife.

[75] Karataeva A.R., Klaassen R.V., Ströder J., Ruiperez-Alonso M., Hjorth J.J., van Nierop P., Spijker S., Mansvelder H.D., Smit A.B. (2014). C-terminal interactors of the AMPA receptor auxiliary subunit Shisa9. PLoS ONE.

[76] Kunde S.A., Rademacher N., Zieger H., Shoichet S.A. (2017). Protein kinase C regulates AMPA receptor auxiliary protein Shisa9/CKAMP44 through interactions with neuronal scaffold PICK1. FEBS Open Bio.

[77] von Engelhardt J., Mack V., Sprengel R., Kavenstock N., Li K.W., Stern-Bach Y., Smit A.B., Seeburg P.H., Monyer H. (2010). CKAMP44: A brain-specific protein attenuating short-term synaptic plasticity in the dentate gyrus. Science.

[78] Khodosevich K., Jacobi E., Farrow P., Schulmann A., Rusu A., Zhang L., Sprengel R., Monyer H., von Engelhardt J. (2014). Coexpressed auxiliary subunits exhibit distinct modulatory profiles on AMPA receptor function. Neuron.

[79] Gu X., Mao X., Lussier M.P., Hutchison M.A., Zhou L., Hamra F.K., Roche K.W., Lu W. (2016). GSG1L suppresses AMPA receptor-mediated synaptic transmission and uniquely modulates AMPA receptor kinetics in hippocampal neurons. Nat. Commun..

[80] Chen X., Aslam M., Gollisch T., Allen K., von Engelhardt J. (2018). CKAMP44 modulates integration of visual inputs in the lateral geniculate nucleus. Nat. Commun..

[81] Liu J., Li S., Li X., Li W., Yang Y., Guo S., Lv L., Xiao X., Yao Y.G., Guan F. (2021). Genome-wide association study followed by trans-ancestry meta-analysis identify 17 new risk loci for schizophrenia. BMC Med..

[82] Shafik A.M., Zhang F., Guo Z., Dai Q., Pajdzik K., Li Y., Kang Y., Yao B., Wu H., He C. (2021). N6-methyladenosine dynamics in neurodevelopment and aging, and its potential role in Alzheimer’s disease. Genome Biol..

[83] Rudnitskaya E.A., Kozlova T.A., Burnyasheva A.O., Tarasova A.E., Pankova T.M., Starostina M.V., Stefanova N.A., Kolosova N.G. (2020). Features of Postnatal Hippocampal Development in a Rat Model of Sporadic Alzheimer’s Disease. Front. Neurosci..

[84] Boccardi V., Murasecco I., Mecocci P. (2019). Diabetes drugs in the fight against Alzheimer’s disease. Ageing Res. Rev..

[85] Ebrahimpour S., Zakeri M., Esmaeili A. (2020). Crosstalk between obesity, diabetes, and alzheimer’s disease: Introducing quercetin as an effective triple herbal medicine. Ageing Res. Rev..

[86] Biessels G.J., Nobili F., Teunissen C.E., Simó R., Scheltens P. (2020). Understanding multifactorial brain changes in type 2 diabetes: A biomarker perspective. Lancet Neurol..

[87] Biessels G.J., Whitmer R.A. (2020). Cognitive dysfunction in diabetes: How to implement emerging guidelines. Diabetologia.

[88] Lanni C., Masi M., Racchi M., Govoni S. (2021). Cancer and Alzheimer’s disease inverse relationship: An age-associated diverging derailment of shared pathways. Mol. Psychiatry.

[89] Horwitz T., Lam K., Chen Y., Xia Y., Liu C. (2019). A decade in psychiatric GWAS research. Mol. Psychiatry.

[90] Lin C.W., Chang L.C., Ma T., Oh H., French B., Puralewski R., Mathews F., Fang Y., Lewis D.A., Kennedy J.L. (2020). Older molecular brain age in severe mental illness. Mol. Psychiatry.

[91] Dominy S.S., Lynch C., Ermini F., Benedyk M., Marczyk A., Konradi A., Nguyen M., Haditsch U., Raha D., Griffin C. (2019). Porphyromonas gingivalis in Alzheimer’s disease brains: Evidence for disease causation and treatment with small-molecule inhibitors. Sci. Adv..

[92] de Oliveira Araújo R., Villoria G.E.M., Luiz R.R., Esteves J.C., Leão A.T.T., Feres-Filho E.J. (2021). Association between periodontitis and Alzheimer’s disease and its impact on the self-perceived oral health status: A case-control study. Clin. Oral Investig..

[93] Dioguardi M., Crincoli V., Laino L., Alovisi M., Sovereto D., Mastrangelo F., Russo L.L., Muzio L.L. (2020). The Role of Periodontitis and Periodontal Bacteria in the Onset and Progression of Alzheimer’s Disease: A Systematic Review. J. Clin. Med..

[94] Liccardo D., Marzano F., Carraturo F., Guida M., Femminella G.D., Bencivenga L., Agrimi J., Addonizio A., Melino I., Valletta A. (2020). Potential Bidirectional Relationship Between Periodontitis and Alzheimer’s Disease. Front. Physiol..

[95] Ide M., Harris M., Stevens A., Sussams R., Hopkins V., Culliford D., Fuller J., Ibbett P., Raybould R., Thomas R. (2016). Periodontitis and Cognitive Decline in Alzheimer’s Disease. PLoS ONE.

[96] Fang X., Tang W., Yang F., Lu W., Cai J., Ni J., Zhang J., Tang W., Li T., Zhang D.F. (2019). A Comprehensive Analysis of the CaMK2A Gene and Susceptibility to Alzheimer’s Disease in the Han Chinese Population. Front. Aging Neurosci..

[97] Vázquez-Higuera J.L., Mateo I., Sánchez-Juan P., Rodríguez-Rodríguez E., Pozueta A., Calero M., Dobato J.L., Frank-García A., Valdivieso F., Berciano J. (2011). Genetic variation in the tau kinases pathway may modify the risk and age at onset of Alzheimer’s disease. J. Alzheimer’s Dis..

[98] Arezoumandan S., Cai X., Kalkarni P., Davis S.A., Wilson K., Ferris C.F., Cairns N.J., Gitcho M.A. (2021). Hippocampal neurobiology and function in an aged mouse model of TDP-43 proteinopathy in an APP/PSEN1 background. Neurosci. Lett..

[99] Jian X.Q., Wang K.S., Wu T.J., Hillhouse J.J., Mullersman J.E. (2011). Association of ADAM10 and CAMK2A polymorphisms with conduct disorder: Evidence from family-based studies. J. Abnorm. Child Psychol..

[100] Zhu Y., Ding X., She Z., Bai X., Nie Z., Wang F., Wang F., Geng X. (2020). Exploring Shared Pathogenesis of Alzheimer’s Disease and Type 2 Diabetes Mellitus via Co-expression Networks Analysis. Curr. Alzheimer Res..

[101] Lyons C.E., Zhou X., Razzoli M., Chen M., Xia W., Ashe K., Zhang B., Bartolomucci A. (2021). Lifelong chronic psychosocial stress induces a proteomic signature of Alzheimer’s disease in wildtype mice. Eur. J. Neurosci..

[102] Bereczki E., Branca R.M., Francis P.T., Pereira J.B., Baek J.H., Hortobágyi T., Winblad B., Ballard C., Lehtiö J., Aarsland D. (2018). Synaptic markers of cognitive decline in neurodegenerative diseases: A proteomic approach. Brain.

[103] Jacob C.P., Koutsilieri E., Bartl J., Neuen-Jacob E., Arzberger T., Zander N., Ravid R., Roggendorf W., Riederer P., Grünblatt E. (2007). Alterations in expression of glutamatergic transporters and receptors in sporadic Alzheimer’s disease. J. Alzheimers Dis..

[104] Grizenkova J., Akhtar S., Collinge J., Lloyd S.E. (2010). The retinoic acid receptor beta (Rarb) region of Mmu14 is associated with prion disease incubation time in mouse. PLoS ONE.

[105] Suszyńska-Zajczyk J., Luczak M., Marczak L., Jakubowski H. (2014). Hyperhomocysteinemia and bleomycin hydrolase modulate the expression of mouse brain proteins involved in neurodegeneration. J. Alzheimer’s Dis..

[106] Hawi Z., Cummins T.D., Tong J., Johnson B., Lau R., Samarrai W., Bellgrove M.A. (2015). The molecular genetic architecture of attention deficit hyperactivity disorder. Mol. Psychiatry.

[107] Wang Y., Guo R., Chen B., Rahman T., Cai L., Li Y., Dong Y., Tseng G.C., Fang J., Seney M.L. (2020). Cocaine-induced neural adaptations in the lateral hypothalamic melanin-concentrating hormone neurons and the role in regulating rapid eye movement sleep after withdrawal. Mol. Psychiatry.

[108] Gray A.L., Hyde T.M., Deep-Soboslay A., Kleinman J.E., Sodhi M.S. (2015). Sex differences in glutamate receptor gene expression in major depression and suicide. Mol. Psychiatry.

[109] Elia J., Gai X., Xie H.M., Perin J.C., Geiger E., Glessner J.T., D’Arcy M., deBerardinis R., Frackelton E., Kim C. (2010). Rare structural variants found in attention-deficit hyperactivity disorder are preferentially associated with neurodevelopmental genes. Mol. Psychiatry.

[110] Ryan N.M., Lihm J., Kramer M., McCarthy S., Morris S.W., Arnau-Soler A., Davies G., Duff B., Ghiban E., Hayward C. (2018). DNA sequence-level analyses reveal potential phenotypic modifiers in a large family with psychiatric disorders. Mol. Psychiatry.

[111] Smith L.N., Jedynak J.P., Fontenot M.R., Hale C.F., Dietz K.C., Taniguchi M., Thomas F.S., Zirlin B.C., Birnbaum S.G., Huber K.M. (2014). Fragile X mental retardation protein regulates synaptic and behavioral plasticity to repeated cocaine administration. Neuron.

[112] Devon R.S., Anderson S., Teague P.W., Muir W.J., Murray V., Pelosi A.J., Blackwood D.H., Porteous D.J. (2001). The genomic organisation of the metabotropic glutamate receptor subtype 5 gene, and its association with schizophrenia. Mol. Psychiatry.

[113] Shin S., Kwon O., Kang J.I., Kwon S., Oh S., Choi J., Kim C.H., Kim D.G. (2015). mGluR5 in the nucleus accumbens is critical for promoting resilience to chronic stress. Nat. Neurosci..

[114] Skafidas E., Testa R., Zantomio D., Chana G., Everall I.P., Pantelis C. (2014). Predicting the diagnosis of autism spectrum disorder using gene pathway analysis. Mol. Psychiatry.

[115] Sathe G., Mangalaparthi K.K., Jain A., Darrow J., Troncoso J., Albert M., Moghekar A., Pandey A. (2020). Multiplexed Phosphoproteomic Study of Brain in Patients with Alzheimer’s Disease and Age-Matched Cognitively Healthy Controls. Omics.

[116] Haas L.T., Salazar S.V., Kostylev M.A., Um J.W., Kaufman A.C., Strittmatter S.M. (2016). Metabotropic glutamate receptor 5 couples cellular prion protein to intracellular signalling in Alzheimer’s disease. Brain.

[117] Loh K.H., Stawski P.S., Draycott A.S., Udeshi N.D., Lehrman E.K., Wilton D.K., Svinkina T., Deerinck T.J., Ellisman M.H., Stevens B. (2016). Proteomic Analysis of Unbounded Cellular Compartments: Synaptic Clefts. Cell.

[118] Kim J.A., Kim D., Won S.Y., Han K.A., Park D., Cho E., Yun N., An H.J., Um J.W., Kim E. (2017). Structural Insights into Modulation of Neurexin-Neuroligin Trans-synaptic Adhesion by MDGA1/Neuroligin-2 Complex. Neuron.

[119] Connor S.A., Elegheert J., Xie Y., Craig A.M. (2019). Pumping the brakes: Suppression of synapse development by MDGA-neuroligin interactions. Curr. Opin. Neurobiol..

[120] Connor S.A., Ammendrup-Johnsen I., Chan A.W., Kishimoto Y., Murayama C., Kurihara N., Tada A., Ge Y., Lu H., Yan R. (2016). Altered Cortical Dynamics and Cognitive Function upon Haploinsufficiency of the Autism-Linked Excitatory Synaptic Suppressor MDGA2. Neuron.

[121] Lee K., Kim Y., Lee S.J., Qiang Y., Lee D., Lee H.W., Kim H., Je H.S., Südhof T.C., Ko J. (2013). MDGAs interact selectively with neuroligin-2 but not other neuroligins to regulate inhibitory synapse development. Proc. Natl. Acad. Sci. USA.

[122] Ceylan H. (2021). Integrated Bioinformatics Analysis to Identify Alternative Therapeutic Targets for Alzheimer’s Disease: Insights from a Synaptic Machinery Perspective. J. Mol. Neurosci..

[123] Jiang H., Jia J. (2009). Association between NR2B subunit gene (GRIN2B) promoter polymorphisms and sporadic Alzheimer’s disease in the North Chinese population. Neurosci. Lett..

[124] Chen C., Li X., Wang T., Wang H.H., Fu Y., Zhang L., Xiao S.F. (2010). Association between NMDA receptor subunit 2b gene polymorphism and Alzheimer’s disease in Chinese Han population in Shanghai. Neurosci. Bull..

[125] Andreoli V., De Marco E.V., Trecroci F., Cittadella R., Di Palma G., Gambardella A. (2014). Potential involvement of GRIN2B encoding the NMDA receptor subunit NR2B in the spectrum of Alzheimer’s disease. J. Neural. Transm..

[126] Neff R.A., Wang M., Vatansever S., Guo L., Ming C., Wang Q., Wang E., Horgusluoglu-Moloch E., Song W.M., Li A. (2021). Molecular subtyping of Alzheimer’s disease using RNA sequencing data reveals novel mechanisms and targets. Sci. Adv..

[127] Chu C.S., Li C.T., Brunoni A.R., Yang F.C., Tseng P.T., Tu Y.K., Stubbs B., Carvalho A.F., Thompson T., Rajji T.K. (2021). Cognitive effects and acceptability of non-invasive brain stimulation on Alzheimer’s disease and mild cognitive impairment: A component network meta-analysis. J. Neurol. Neurosurg. Psychiatry.

[128] Segel M., Lash B., Song J., Ladha A., Liu C.C., Jin X., Mekhedov S.L., Macrae R.K., Koonin E.V., Zhang F. (2021). Mammalian retrovirus-like protein PEG10 packages its own mRNA and can be pseudotyped for mRNA delivery. Science.

[129] Nagy C., Maitra M., Tanti A., Suderman M., Théroux J.F., Davoli M.A., Perlman K., Yerko V., Wang Y.C., Tripathy S.J. (2020). Single-nucleus transcriptomics of the prefrontal cortex in major depressive disorder implicates oligodendrocyte precursor cells and excitatory neurons. Nat. Neurosci..

