# Supplementary material for: Single-Cell Transcriptomic Profiling Reveals Regional Differences in the Prefrontal and Entorhinal Cortex of Alzheimer’s Disease Brain
Source: Int J Mol Sci. 2025 May 19;26(10):4841. doi: 10.3390/ijms26104841 (PMC12112128; doi:10.3390/ijms26104841)
Supplement: Supplementary file 1 [file ijms-26-04841-s001.zip › Supplementary materials.pdf]

Supplementary figures

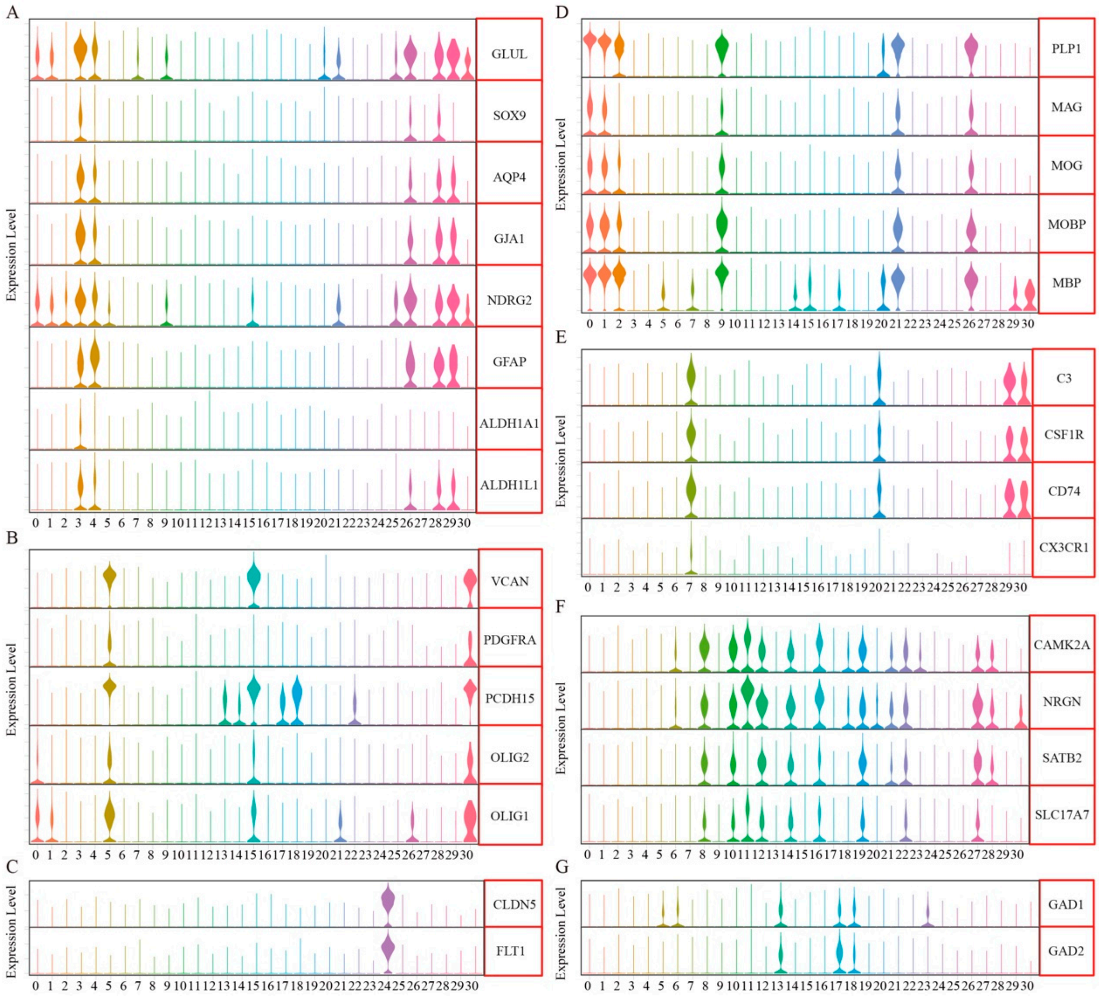

Fig. S1 Distribution of cell-specific marker genes. A, Astro specific marker genes. **B,** OPC specific marker genes. **C,** Specific marker genes of Endo. **D,** Oligo-specific marker gene expression. **E,** Micro specific marker genes. **F,** Specific marker genes of ExN. **G,** Specific marker genes of InN.

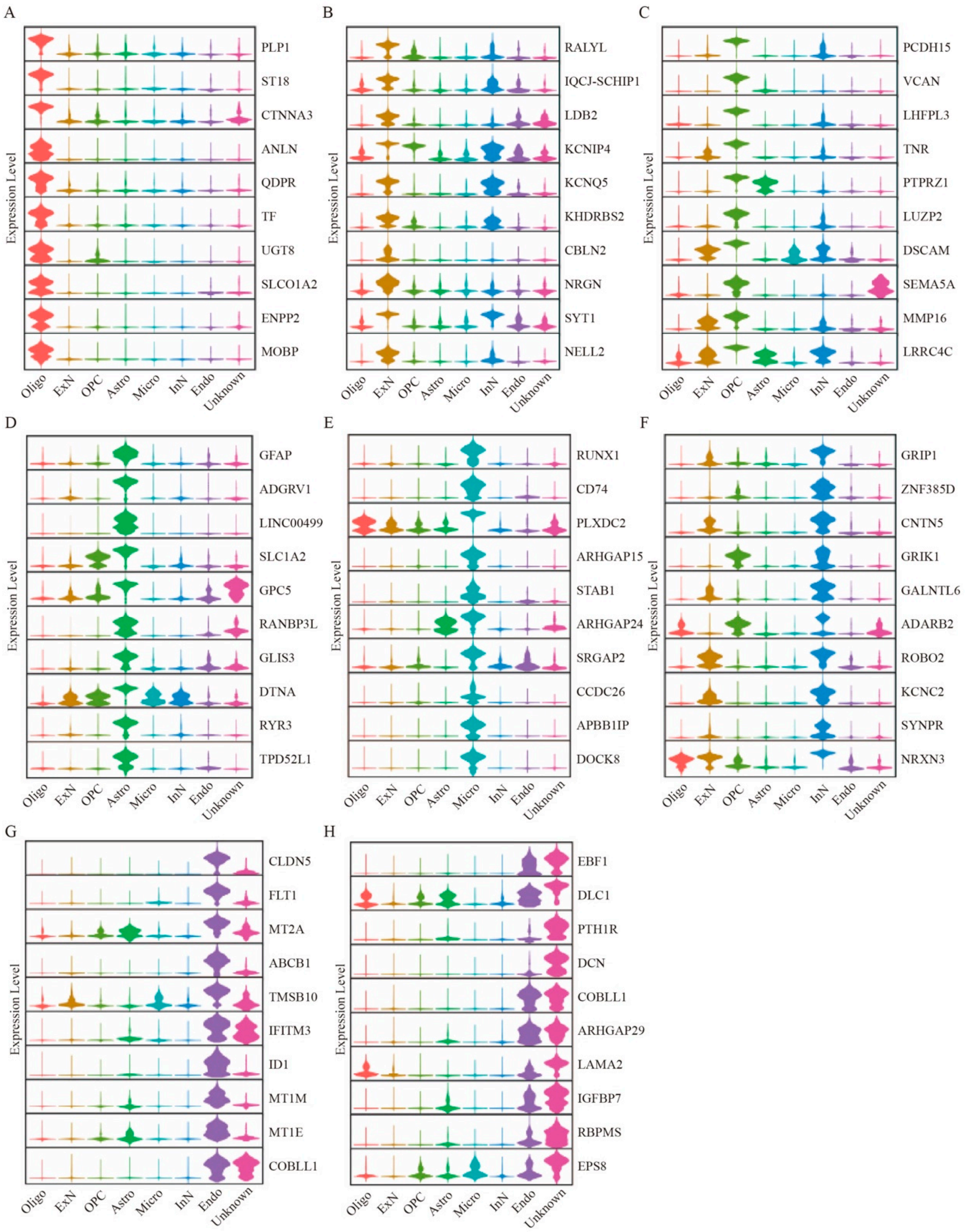

Fig. S2 Gene expression of top 10 DEGs in eight cell types. Violin plots show specific marker genes expression of Oligo (**A**), ExN (**B**), OPC (**C**), Astro (**D**), Micro (**E**), InN (**F**), Endo (**G**), and Unknown cell (**H**).

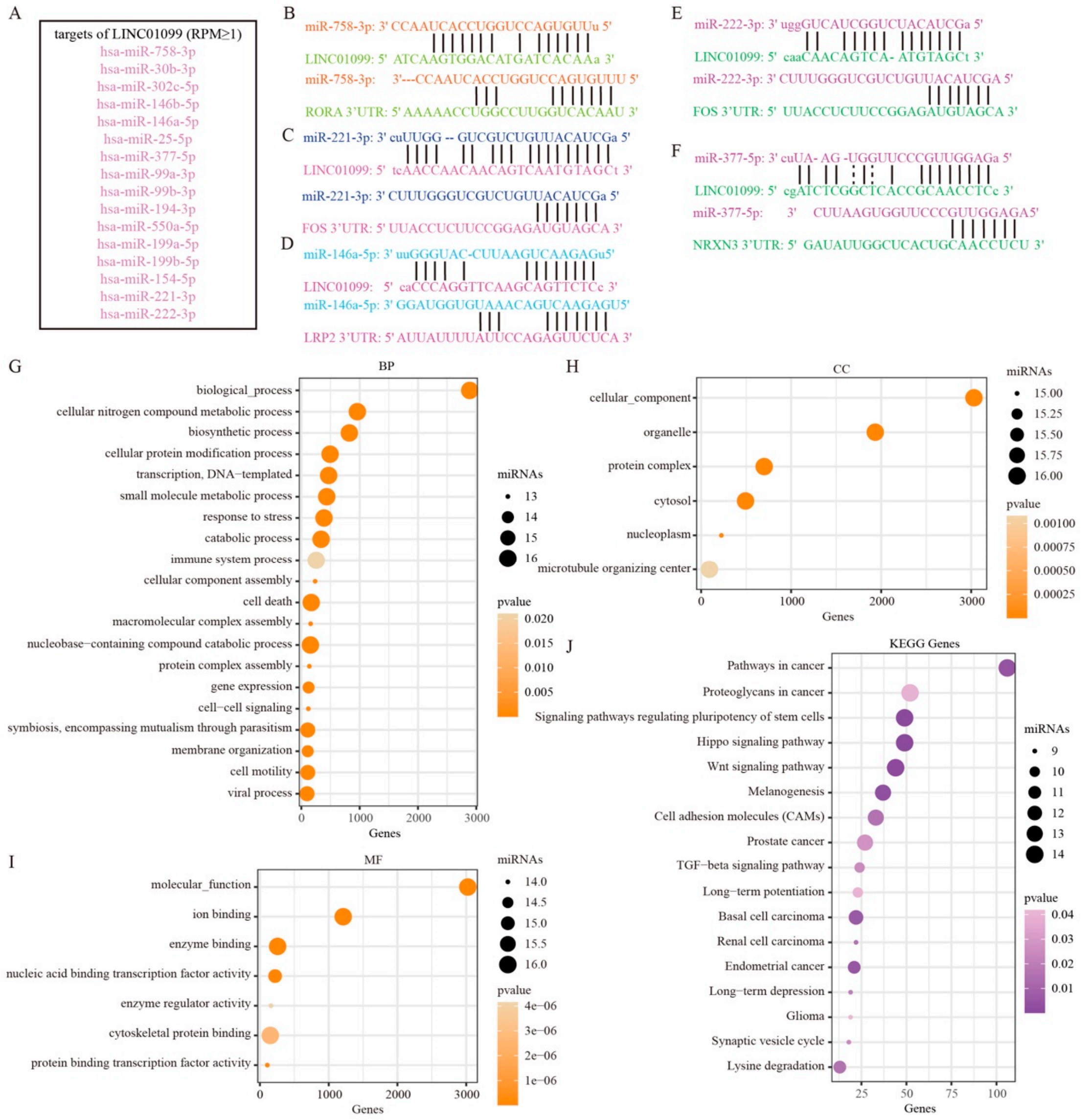

Fig. S3 Targeted miRNAs of *LINC01099* and enrichment analysis. A, Sixteen targeted miRNAs of *LINC01099* (RPM ≥ 1) are shown. **B,** Potential binding sites of *LINC01099* to *miR-758-3p*, *miR-758-3p* to *RORA*. **C,** Potential binding sites of *LINC01099* to *miR-221-3p*, *miR-221-3p* to *FOS*. **D,** Potential binding sites of *LINC01099* to *miR-146a-5p*, *miR-146a-5p* to *LRP2*. **E,** Potential binding sites of *LINC01099* to *miR-222-3p*, *miR-222-3p* to *FOS*. **F,** Potential binding sites of *LINC01099* to *miR-377-5p*, *miR-377-5p* to *NRXN3*. **G-I,** GO enrichment analysis on 16 miRNAs, including BP (**G**), CC (**H**), and MF (**I**), respectively. **J,** KEGG enrichment analysis for 16 miRNAs.

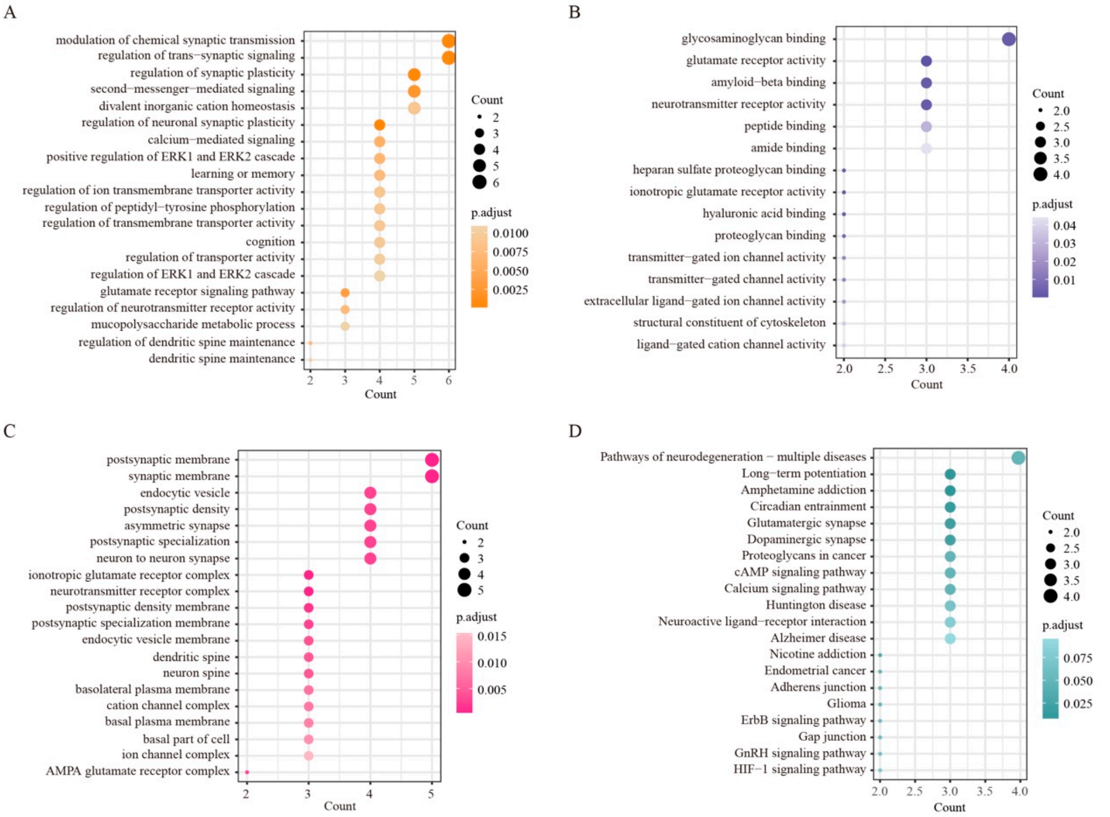

Fig. S4 GO and KEGG signaling pathway enrichment analysis for cellular key regulatory genes. A-C, GO enrichment analysis of key regulatory genes in the 8 cell types, BP **(A),** MF **(B)**, and CC **(C)**. **D,** Analysis of KEGG signaling for the crucial regulatory genes in the 8 cell types.

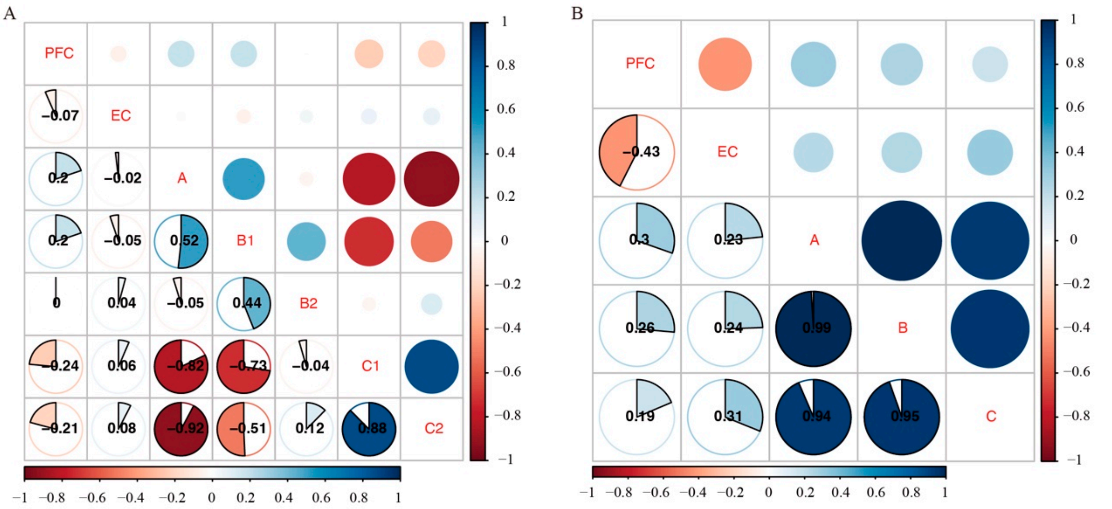

Fig. S5 The comparative analysis between snRNA-seq and the results of Neff RA et al. A, Correlation analysis between DEGs of snRNA-seq and DEGs of five AD subtypes (A, B1, B2, C1, C2) reported by Neff RA et al. **B,** Correlation analysis between DEGs of snRNA-seq and DEGs of three major classes (A, B, C) as reported by Neff RA et al.

Supplementary tables

Table S1: Patient pathology and clinical history. Clinical traits of the individuals involved in this study, including age, gender, post mortem interval (PMI), diagnosis, ApoE genotype and amyloid pathology.

Table S2: The overlapping DEGs for PFC and EC.

Table S3: The target genes of these miRNAs were subsequently predicted using miRDB, miRTarBase, Targetscan.

Table S4: The overlapping DEGs of the bulk RNA-seq and snRNA-seq datasets.

Table S5: The PPI analysis for the overlap DEGs of the bulk RNA-seq and snRNA-seq datasets.

Table S6: The key regulatory genes in the 8 cell types.

Table S7: The GO (BP) enrichment analysis for key regulatory genes.

Table S8: The GO (MF) enrichment analysis for key regulatory genes.

Table S9: The GO (CC) enrichment analysis for key regulatory genes.

Table S10: The KEGG enrichment analysis for key regulatory genes.
